# A global assessment of invasive plant impacts on resident species, communities and ecosystems: the interaction of impact measures, invading species' traits and environment

**DOI:** 10.1111/j.1365-2486.2011.02636.x

**Published:** 2012-05

**Authors:** Petr Pyšek, Vojtěch Jarošík, Philip E Hulme, Jan Pergl, Martin Hejda, Urs Schaffner, Montserrat Vilà

**Affiliations:** * Institute of Botany, Department of Invasion Ecology, Academy of Sciences of the Czech RepublicCZ-252 43, Průhonice, Czech Republic; † Department of Ecology, Faculty of Science, Charles University PragueViničná 7, CZ-128 44, Prague, Czech Republic; ‡ The Bio-Protection Research Centre, Lincoln UniversityPO Box 84, Canterbury, New Zealand; § Institute of Ecology and Evolution, University of BernCH-3012, Bern, Switzerland; ¶ CABI Europe-Switzerland, 1 Rue des GrillonsCH-2800, Delémont, Switzerland; | Estación Biológica de Doñana (EBD-CSIC), Avda. Américo Vespucio s/nIsla de la Cartuja, E-41092, Sevilla, Spain

**Keywords:** biome, invasive plants, islands, prediction, resident biota, soil resources, species diversity, species traits

## Abstract

With the growing body of literature assessing the impact of invasive alien plants on resident species and ecosystems, a comprehensive assessment of the relationship between invasive species traits and environmental settings of invasion on the characteristics of impacts is needed. Based on 287 publications with 1551 individual cases that addressed the impact of 167 invasive plant species belonging to 49 families, we present the first global overview of frequencies of significant and non-significant ecological impacts and their directions on 15 outcomes related to the responses of resident populations, species, communities and ecosystems. Species and community outcomes tend to decline following invasions, especially those for plants, but the abundance and richness of the soil biota, as well as concentrations of soil nutrients and water, more often increase than decrease following invasion. Data mining tools revealed that invasive plants exert consistent significant impacts on some outcomes (survival of resident biota, activity of resident animals, resident community productivity, mineral and nutrient content in plant tissues, and fire frequency and intensity), whereas for outcomes at the community level, such as species richness, diversity and soil resources, the significance of impacts is determined by interactions between species traits and the biome invaded. The latter outcomes are most likely to be impacted by annual grasses, and by wind pollinated trees invading mediterranean or tropical biomes. One of the clearest signals in this analysis is that invasive plants are far more likely to cause significant impacts on resident plant and animal richness on islands rather than mainland. This study shows that there is no universal measure of impact and the pattern observed depends on the ecological measure examined. Although impact is strongly context dependent, some species traits, especially life form, stature and pollination syndrome, may provide a means to predict impact, regardless of the particular habitat and geographical region invaded.

## Introduction

Despite an increasing body of information addressing the impacts of invasive plants, much of the literature to date aims to quantitatively describe the pattern of ecological impacts and the putative mechanisms acting in individual case studies rather than develop synthesis (Levine *et al*., 2003). Such a synthesis is essential in the face of the increasing frequency and magnitude of plant introductions and the mounting pressure on policymakers to regulate and mitigate this component of global change (Lodge *et al*., 2006; Hulme *et al*., 2009b). Recent attempts to review plant impact studies using meta-analyses have either been focused on specific ecosystems (Gaertner *et al*., 2009) or types of impact (Liao *et al*., 2007; Powell *et al*., 2011; Vilà *et al*., 2011). As a consequence, we still lack a broad quantitative synthesis of how impacts vary in relation to the attributes of recipient ecosystems and of the invading plants themselves (Parker *et al*., 1999; Thiele *et al*., 2010a, b; Hulme, 2012). For example, most research on species traits seeks to identify those characteristics that determine plant invasiveness rather than impact (Daehler, 2003; Pyšek & Richardson, 2007; Pyšek *et al*., 2009; Van Kleunen *et al*., 2010). Only in the case of nitrogen-fixing taxa, has an attempt been made to link a particular plant trait with subsequent impact and vulnerability of recipient ecosystems (Ehrenfeld, 2003, 2010; Liao *et al*., 2007). However, beyond this example, it is not yet possible to generalize as to whether or not traits that contribute to invasiveness are also responsible for causing impacts. This is because studies and reviews conducted so far have largely focused on the quantification and description of impacts rather than on relating it to species traits. Knowledge of which species traits determine impact, and how they might be dependent on environmental settings, such as habitat and biome, would certainly assist in developing new tools for assessing not just the likelihood but also the consequences of plant invasions (Daehler & Virtue, 2010; Hulme, 2011, 2012). Moreover, the recent critique of invasion studies emphasizes not only a need to move away from predicting naturalization but to link this to species impact (Davis *et al*., 2011; but see Lambertini *et al*., 2011; Simberloff *et al*., 2011).

The impact of invasive plant species on resident species, communities and ecosystems is manifest in various ways. By reducing species richness and abundance of native biota and decreasing their local species diversity, invasions reduce the distinctiveness of biological communities at various spatial scales (Olden & Poff, 2003; Sax & Gaines, 2003; Winter *et al*., 2009). Other impacts include effects on the genetic variation of native populations via hybridization (Vilà *et al*., 2000), and disruptions of mutualistic networks such as pollination and dispersal (Traveset & Richardson, 2006; Schweiger *et al*., 2010). Some invasive plants change habitat and ecosystem functioning (Richardson *et al*., 2000; Hulme, 2007; Vilà *et al*., 2009, 2011) to the extent of having impacts upon ecosystem services and human well-being (Pejchar & Mooney, 2009; Pyšek & Richardson, 2010). It is also important to emphasize that such impacts may not be perceived as negative (Schlaepfer *et al*., 2011). For individual species, the significance of impacts can vary in relation to habitat type and, even for the same habitat, significant variations in both the direction and magnitude of impacts can be found among regions (Vilà *et al*., 2006). Such evidence emphasizes the need to simultaneously assess the role of species traits as well as the invasion context when attempting to predict impacts of invasive plants.

In response to the absence of general synthesis of plant invasion impacts, herein we undertake a comprehensive, evidence-based assessment of the role of species traits and environmental context on the consequences of plant invasions. We focus on ecological impacts rather than on economic or human welfare costs, since information on the former is more widely available. In contrast to studies that have adopted meta-analytical approaches to synthesis to avoid problems with vote counting (see e.g. Hedges & Olkin, 1985; Gurevitch & Hedges, 1999, 2001), we applied data mining methods due to three clear advantages. First, invasive plant impacts vary in both their magnitude and direction. Thus the calculation of mean effect sizes may fail to detect significant trends where both increases and decreases of a response variable occur, since they may on average cancel themselves out. This arises because the crucial effects of ecological impacts can often appear not as main effects, but in interactions with other effects. Second, unlike meta-analyses, data mining tools provide the opportunity to examine complex interactions among variables which we expect to be important given the context dependence of many observed impacts. Third, meta-analytical approach requires *a priori* defined hypotheses to be compared using *a priori* selected explanatory variables. However, the data mining techniques allow predictions to be derived from the data and identify the most important explanatory variables by screening a large number of candidate variables without requiring any assumptions about the form of the relationships between explanatory variables and the response variable, and without *a priori* formulated hypotheses (Hochachka *et al*., 2007).

We present the first global overview of the frequencies of significant and non-significant impacts and their directions on a broad range of characteristics related to resident species, their populations, and the communities in which they occur as well as ecosystem processes. By evaluating the direct and interactive effects of plant species traits in various environmental and geographical situations (e.g. invaded habitat, geographical region and biome), on whether or not the invasions result in significant impacts, we aim to provide quantitative insights into the predictability of plant invasions impacts.

## Materials and methods

### Data collation

Data were gathered following standard evidence-based protocols that included searching the primary literature and subsequent snowballing to older or secondary literature as well as establishing objective criteria for including a study in the subsequent analysis. We searched for relevant articles on the ISI Web of Knowledge (http://www.apps.webofknowledge.com) database on 11 March 2009 with no restriction on publication year, using the following search term combinations: (plant invader OR exotic plant OR alien plant OR plant invasion*) AND (impact* OR effect*) AND (community structure* OR diversity* OR ecosystem process* OR competition*). As the next step, we also screened the reference lists from all retrieved articles for other relevant publications that included book chapters and ‘grey’ literature (e.g. papers in local journals, doctoral theses, government reports). As a result, we achieved comprehensive coverage of the literature on alien plant impacts not restricted to that indexed in Web of Science.

This initial screening resulted in 533 publications addressing the impacts of alien plant species. We examined each publication according to the following selection criteria: (1) The study compared invaded and uninvaded plots quantitatively, and statistically tested for the significance of differences in ecological patterns or processes (i.e. of impact) between the two types of plots. (2) As we were interested in relating the traits of the invasive species to their impacts we only selected field studies where the impact could be assigned to a particular species and excluded those referring to impacts of multispecies assemblages. Our final dataset included field studies that were either observational (i.e. comparing non-manipulated invaded and uninvaded sites) or experimental (i.e. based on removal or addition of an invasive plant species) with explicit mention of the identity of the alien plant taxon causing impact; studies from common gardens were excluded because the extent to which their results can be extrapolated to dynamics under natural conditions is debatable. Where the same article examined several alien species, or invasion of a single alien species in several ecosystems, or the impact was measured by using more than one response variable (e.g. effect on both species richness and productivity), we considered these separately as different case studies. When the study investigated the effects of different degrees of invasion (e.g. heavily vs. less invaded sites) or chronosequences (i.e. old vs. recent invasions) we only considered the putative largest contrast. That is, we examined differences between the uninvaded or least invaded, and the most invaded sites, or differences between uninvaded sites and sites with the longest time since invasion.

The screening resulted in almost half the studies being rejected and only 287 studies met the above criteria (Appendix S1 in Supporting Information) that addressed the impact of alien plant species and statistically tested for its significance. The short-listed species can be considered invasive (sensu Richardson *et al*., 2000) since they were both widespread in the study region as well as locally abundant/dominant in the ecosystem targeted for study. Thus for brevity and consistency we use the term ‘invasive plant’ or ‘invader’ as a synonym for invasive alien plant or alien invader. This set of studies yielded data on a number of measures of impact (hereafter referred to as outcomes) for which there was sufficient literature to allow for a quantitative analysis (Table 1). We recorded whether the study concluded that the invasion had affected the given measure of impact significantly or non-significantly (which in the vast majority of cases was at least at *P* < 0.05), and if there were a statistically significant effect, whether it resulted in an increase or decrease of the value of a given measure. In total, the data set included 1551 individual cases of statistically tested impacts of plant invasions. There were 53 studies addressing one case, 66 with 2, 39 with 3, 32 with 4, 19 with 5, 17 with 6 and 61 studies addressed 7 or more cases. Nevertheless for each species × site × outcome we only have one value.

**Table 1:** Overview of the outcomes following alien plant invasions as addressed in the 287 studies considered. Four groups of impact targets were considered: (A) on resident populations, species and communities of plants, (B) on resident populations, species and communities of animals associated with invaded vegetation, (C) on soil characteristics and (D) on fire frequency. The organization level of each outcome is indicated in parentheses: S – measure of impact on species and their populations; C – measure of impact on community characteristics; E – measure of impact on ecosystem characteristics. Note that the impacts 1–8 refer to those on resident biota, impacts 9–15 compare the difference in state of the community/ecosystem prior and after invasion, that is, the latter includes the invading species. For each outcome within each group, total number of cases analysed (in bold), the number of those when invasion caused a significant decrease or increase, and the total number of studies addressing the given outcome in the group is indicated

	A. Plants				B. Animals				C. Soil				D. Fire			
Outcomes	Cases	Decrease	Increase	Studies	Cases	Decrease	Increase	Studies	Cases	Decrease	Increase	Studies	Cases	Decrease	Increase	Studies
**Species level effects**																
1. Survival of resident biota (S)	**25**	19	1	18	**20**	9	3	11								
2. Fecundity of resident biota (S)	**20**	16	1	11	**8**	5	0	6								
3. Activity of resident animals (S)					**18**	11	5	10								
**Community level effects**																
4. Abundance of resident biota (C)	**15**	9	3	12	**85**	28	11	34	**31**	11	11	10				
5. Species richness of resident biota (C)	**95**	59	8	50	**37**	13	1	25	**9**	8	1	5				
6. Species diversity of resident biota (C)	**65**	40	3	29	**25**	9	0	12	**4**	1	2	2				
7. Productivity of resident biota (C)	**105**	51	34	57	**10**	6	1	8	**45**	14	24	26				
8. Cover of resident plants (C)	**27**	16	4	17												
**Ecosystem level effects**																
9. Mineral and nutrient contents in plant tissues (E)	**60**	4	46	12												
10. Mineral content in soil (E)									**242**	22	77	28				
11. Water content in soil (E)									**22**	12	3	16				
12. Nutrient content in soil (E)									**436**	72	196	94				
13. pH of soil (E)									**62**	18	10	35				
14. Rate of litter decomposition (E)									**25**	7	17	18				
15. Fire frequency and intensity (E)													**60**	2	58	35
**Total number of cases**	412	214	100		203	81	21		876	165	341		60	2	58	

Survival includes also mortality (coded in opposite direction so as to have the same effect as survival) and impact on seedling establishment of resident plants. Activity of resident animals includes, for example, nesting activities, pollinator visits etc. Abundance of resident biota includes numbers, density and cover of plants and animals. Productivity was measured as biomass or NPP and also included measures of growth. Nutrient contents in the soil include impacts on C, N, P cycling, soil OM and microbial activity.

### Classification of impacts

A two-step classification of impacts was adopted that cross-tabulated the target with the outcome (Table 1). Targets included: (A) populations, species and communities of plants; (B) populations, species and communities of animals; (C) soil characteristics; and (D) fire regime, representing the only type of disturbance for which enough cases were available for the analysis. We broadly discuss impacts on soil characteristics and fire regime as changes to ecosystem processes. For each of the four targets, one or more outcomes were identified (e.g. change in survival, abundance etc.). A total of 15 outcomes were derived from the studies that could broadly be identified with species, community or ecosystem level effects (Table 1).

The data set included 167 invasive plant species from 49 families (Appendix S2 in Supporting Information; the nomenclature used follows that in the original studies). Where possible, each species was categorized in relation to taxonomic affiliation (genus, family, order, subclass), flowering period (in months), life form (annual herb, perennial herb, annual grass, perennial grass, shrub, tree, vine), pollination system (wind, insect, water, self), presence of thorns/spines, seed size, height, nitrogen fixation, toxicity, dispersal syndrome (wind, water, endozoochory, exozoochory, autochory), and clonal growth. Therefore, we examined traits that might facilitate recruitment (seed size, dispersal syndrome, pollination), competition (height, nitrogen fixation, clonal growth) and resistance to generalist herbivores (spines/thorns, toxicity). The information on the traits of invading plant species was obtained using regional floras, checklists of invasive species, global compendia (e.g. Weber, 2003), internet databases and other sources. For each case study, the following site characteristics were recorded (Table 2): (1) region (Africa, Asia, Australasia, Europe, North America, Pacific, South America); (2) biome (temperate, mediterranean, subtropical, tropical); (3) insularity (island or continent); and (4) habitat (anthropogenic, arid, coastal, grassland, riparian, rocky, shrubland, woodland).

**Table 2:** Distribution of the number of cases analysed (*n* = 1551) according to the characteristics of the invaded site

Site invaded	Africa	Asia	Australia	Europe	North America	Pacific	South America	Total
Region total	107	53	139	327	708	140	77	
Biome invaded								
Temperate	5	2	22	285	556	30	25	925
Mediterranean	90	15	39	42	109		7	302
Subtropical	8	29	48		43	6	7	141
Tropical	4	7	30			104	38	183
Habitat invaded								
Anthropogenic			1	86	3			90
Arid	2	15	3		3			23
Coastal			10	36	22			68
Grassland	15	6	35	87	435	45	34	657
Riparian	6	8	22	36	133	4		209
Rocky			10	36	16			62
Shrubland	85		64	18	53	2	4	226
Woodland	6	26	26	71	180	93	39	441
Insularity								
Island	7	6	13	20		109	18	173
Mainland	100	47	126	307	708	31	59	1378

### Statistical analysis

The significance score (statistically significant or non-significant as tested in the published study) was the response variable, and outcome, taxonomic affiliation, species traits and site characteristics were the explanatory variables. To reveal general factors determining the significance score, data were pooled across all outcomes. As a second step, separate analyses were carried out to reveal the impact on individual outcomes. These analyses were restricted to outcomes for which there was a sufficiently large sample size and that could be logically grouped together based on the direction of impact. They included changes to resident species richness of plants and animals (*n* = 132), community productivity (*n* = 160) and soil resources (mineral, water and nutrient content, *n* = 700). In the first two analyses addressing species richness and productivity an additional binary variable distinguished between impacts on plants or animals. To make the effect of individual species comparable, the significance score for each species was weighted by the number of records of the species in the given analysis, and by the number of species in a published study (if a single study explored the impact of several invasive species on, for example, resident species diversity).

Classification trees were used for analyses, due to their flexibility and robustness, invariance to monotonic transformations of predictor variables, their ability to use combinations of explanatory variables that are either categorical and/or numeric, facility to deal with nonlinear relationships and high-order interactions, and capacity to treat missing data, which was the case for some of our explanatory variables (De'ath & Fabricius, 2000). The trees are nonparametric and unlike parametric linear models, collinearity does not prevent reliable parameter estimates because the method guards against the elimination of variables which are good predictors of the response, and may be ecologically important, but are correlated with other predictors (Cutler *et al*., 2007). The trees were constructed in CART Pro v. 6.0 (Breiman *et al*., 1984; Steinberg & Colla, 1997; Steinberg & Golovnya, 2006) by binary recursive partitioning, using the default ‘Gini’ impurity measure as the splitting index with balanced class weights, assuring that significant and insignificant impacts were treated as equally important for the purpose of achieving classification accuracy. To determine the optimal tree, a sequence of nested trees of decreasing size, each of them being the best of all trees of its size, were constructed, and their resubstitution relative errors were estimated. Tenfold cross-validation was used to obtain estimates of cross-validated relative errors of these trees. These estimates were then plotted against tree size, and the optimal tree chosen based on the one-SE rule, which minimizes cross-validated error within one standard error of the minimum (Breiman *et al*., 1984). Following De'ath & Fabricius (2000), a series of 50 cross-validations were run, and the modal (most likely) single optimal tree chosen for description.

The quality of the chosen tree was evaluated as the overall misclassification rate by comparing the misclassification rate of the optimal tree with the misclassification rate of the null model (De'ath & Fabricius, 2000), and using cross-validated samples (Steinberg & Colla, 1997) as specificity (i.e. the ability of the model to predict that the impact is not significant when it is not) and sensitivity (the ability of the model to predict that the impact is significant when it is) (Bourg *et al*., 2005). The optimal tree was represented graphically, with the root standing for undivided data at the top, and the terminal nodes, describing the most homogeneous groups of data, at the bottom of the hierarchy. The quality of each split was expressed by its improvement value, corresponding to the overall misclassification rate at the node, with high scores of improvement values corresponding to splits of high quality. In graphical representations, the vertical depth of each node was expressed as proportional to its improvement value. Vertical depth of each node thus represented a value similar to explained variance in a linear model. Surrogates of each split, describing splitting rules that closely mimicked the action of the primary split, were assessed and ranked according to their association values, with the highest possible value 1.0 corresponding to the surrogate producing exactly the same split as the primary split. Using weighted values, which were expressed for each species as fractions decreasing with increasing number of replicates of the species in the analysis, the minimum size of each terminal node was limited to one.

Because categorical explanatory variables with many levels have higher splitting power than continuous variables, to prevent any inherent advantage these variables might have over continuous variables, penalization rules for categorical variables with many levels (Steinberg & Colla, 1997, p. 88) were applied. Similarly, explanatory variables with missing values have an advantage as splitters. Consequently, these variables were first penalized in proportion to the degree to which their values were missing, and then treated by back-up rules using surrogates that closely mimicked the action of the missing primary splitters (Steinberg & Colla, 1997). Classification trees cannot properly handle nested designs such as hierarchical taxonomic level, thus to take into account that related taxa can have similar impact (e.g. Harvey & Pagel, 1991), the trees were first constructed including only the highest taxonomic level (class) and only then including each lower taxonomic level (Jarošík, 2011). This hierarchical treatment of taxonomy could reveal at which taxonomic level related species share traits that might similarly affect the impact.

There are several pitfalls in the synthesis of data drawn from an unplanned, non-orthogonal and heterogeneous source such as published studies. Because the same species may have been examined across different impacts in a single study or for the same impact across different studies there is a danger that well studied species may have undue influence on the results. Similarly, some studies may have undue bias in the outcomes observed if they examined many invasive species, a large number of sites and/or several different impacts. We attempted to address these biases in two ways. First, for each class of impact the significance score for each species was weighted by the number of records of the species included in the analysis. Second, the significance score was also weighted by the number of species included in a published study. These approaches prevented pseudoreplication (Hurlbert, 1984) as they ensured that we did not treat different cases from a single study and multiple cases of the same invasive species in the dataset as independent data points. A third source of bias may also arise from specific authors having an undue influence on outcomes if they contributed to multiple studies whether on the same or on different species. Although we assume most authors undertook their research objectively, there is little evidence that any one author might sway the patterns observed. For example, examination of the first authors of the 287 studies shows that only six researchers were first authors of three studies, 22 of 2 studies and 225 were first authors of only one study.

## Results

### Frequency and direction of significant and non-significant impacts

**Figure 1:**
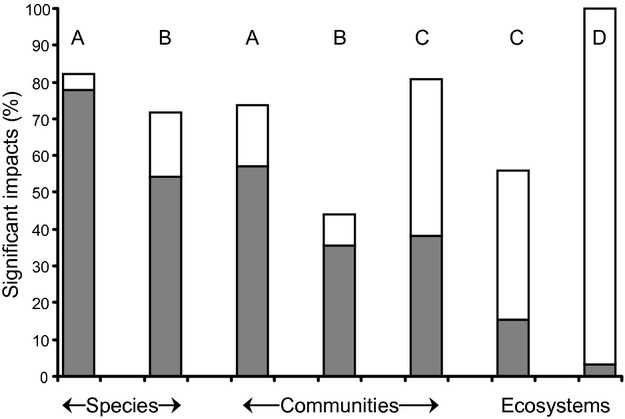
Proportion of impacts causing decrease (shaded) or increase (unshaded) in the outcome measures, summarized according to impact targets (A, species and communities of resident plants; B, species and communities of resident animals associated with invaded vegetation; C, soil characteristics; D, fire regime) and organizational levels of species, communities and ecosystems (See Table 1). The height of the bar corresponds to the percentage of significant impacts. Note that ecosystem effects in this plot refer to soil characteristics excluding pH where the direction of change has a different meaning.

In the majority of cases studied, the presence of an invasive plant caused a significant change in the observed outcomes. For pooled data across the 1551 cases, the impact was significant in 982 cases (63.3%). The proportion of significant impacts was highest on outcome associated with plants (76.2% of 412 cases), followed by soil (57.8% of 876 cases) and then animals (50.2% of 203 cases) although the impact on the fire regime was always significant. Some broad patterns can be outlined in terms of the direction of significant impacts (Fig. 1). First, plant invasions had consistently more frequent impacts on plant than animal outcomes, with the majority of studies reporting decreases at both the species- and community levels. Second, in contrast to plant and animal outcomes, soil attributes tended to increase following invasion at both community and ecosystem-levels. Third, plant invasions overwhelmingly tended to increase ecosystem responses related to fire frequency and intensity.

### General factors determining the significance of impacts

**Figure 2:**
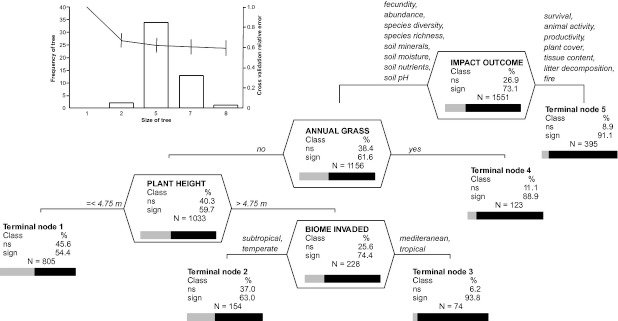
Classification tree analysis of the probability of significant ▪ or non-significant □ impacts on plants, animals, soil and fire frequency. Each node (polygon labelled with splitting variable name) and terminal node with node number includes a table with the impact score (significant or non-significant) and% of these cases for each class (weighted values). Below the table is the total number of cases (N, unweighted) and graphical representation of the percentage of significant and non-significant cases in each class (horizontal bar based on weighted numbers). At each node for each splitting variable, there is a split criterion on its left and right side. Vertical depth of each node is proportional to its improvement value that corresponds to explained variance at the node. Overall misclassification rate of the optimal tree is 33.3%, compared to 50% for the null model; specificity (ability to predict that the impact is not significant when it is not) = 0.78; sensitivity (ability to predict that the impact is significant when it is) = 0.59. Inset: Cross-validation processes for the selection of the optimal regression tree. The line shows a single representative 10-fold cross-validation of the most frequent (modal) optimal tree with standard error (SE) estimate of each tree size. Bar charts are the numbers of the optimal trees of each size (Frequency of tree) selected from a series of 50 cross-validations based on the one-SE rule which minimizes the cross validated error within one standard error of the minimum. The most frequent (modal) tree has five terminal nodes.

For pooled data, invasion was more likely to exert a significant impact on the survival of resident biota, activity of animals, community productivity and cover, the mineral and nutrients content in plant tissues and fire frequency (Fig. 2). The remaining outcomes, mostly related to species abundance, diversity, richness and fecundity of resident biota, and to soil attributes, were more likely to be significantly impacted if the invasive species was an annual grass (significant impacts in 88.9% of cases, Terminal node 4). When the impact was caused by other plant life forms, it was more likely significant when they were taller than 4.8 m (e.g. mostly trees) and the invasion occurred in mediterranean or tropical regions (significant impact in 93.8% of cases, Terminal node 3). In contrast, short-statured plants other than grasses were least likely to exert significant impacts on outcomes related to species diversity and soil attributes (only significant in 54.4% of cases, Terminal node 1).

### Factors determining the significance of impacts on individual outcomes

**Figure 3:**
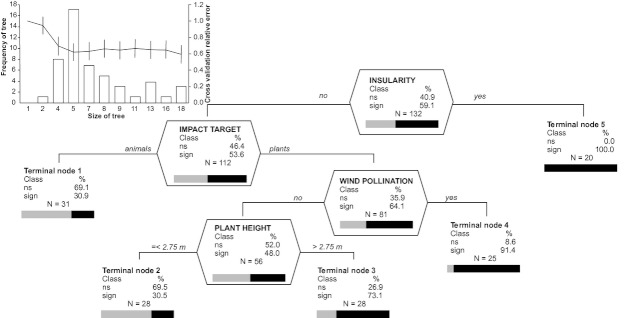
Classification tree analysis of the probability of significant ■ or non-significant □ impacts on species richness. Overall misclassification rate of the optimal tree is 21.5%, compared to 50% for the null model; specificity (ability to predict that the impact is not significant when it is not) = 0.77; sensitivity (ability to predict that the impact is significant when it is) = 0.61. Inset: Cross validation processes for the selection of the optimal regression tree. The line shows a single representative 10-fold cross-validation of the most frequent (modal) optimal tree with standard error (SE) estimate of each tree size. Bar charts are the numbers of the optimal trees of each size (Frequency of tree) selected from a series of 50 cross-validations based on the one-SE rule which minimizes the cross-validated error within one standard error of the minimum. The most frequent (modal) tree has five terminal nodes. Otherwise as in Fig. 2.

The analysis on pooled data revealed that the frequency of significant impacts overwhelmingly depended on the particular outcome examined thus, where sample sizes allowed, we further analysed the three most influential outcomes (species richness, community productivity and soil resources) separately. Regardless of other environmental settings of the invasion and of traits of the invading species, impact on species richness was always significant on islands (significant impact in 100% of cases, Terminal node 5, Fig. 3). On mainlands, richness of resident animals was generally unlikely to be significantly impacted (significant impact in only 30.9% of cases, Terminal node 1). Resident plant communities on mainlands were most likely to suffer from a significant impact on richness if the invasive plant was wind pollinated (91.4% of cases, Terminal node 4). Invasive plants pollinated by means other than wind only exerted such significant impact if they were taller than 2.8 m (in 73.1% of cases; Terminal node 3), whereas those that were shorter than this threshold were the least likely to cause significant impact of all groups identified by the classification tree (30.5% of cases, Terminal node 2).

**Figure 4:**
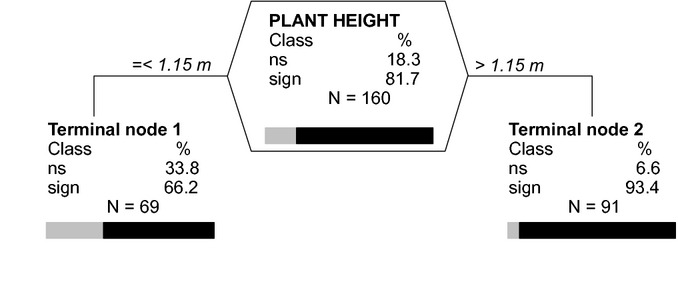
Classification tree analysis of the probability of significant ■ or non-significant □ impacts on the productivity of the native biota. Overall misclassification rate of the optimal tree is 32.1%, compared to 50% for the null model; specificity (ability to predict that the impact is not significant when it is not) = 0.74; sensitivity (ability to predict that the impact is significant when it is) = 0.65. The most frequent (modal) tree always had two terminal nodes. Otherwise as in Fig. 2.

The significance of impact on productivity of the resident plant and animal communities and on plant community cover depended only on the height of the invading plant (Fig. 4). Species taller than 1.2 m exerted significant impact in 93.4% of cases (Terminal node 2), whereas those that were shorter only in 66.2% (Terminal node 1). The analysis of impact on soil nutrient, mineral and water contents split the 700 cases analysed into two groups based on the membership of invading plant species to individual genera (results not shown) and did not reveal any effect of plant species traits and site characteristics.

## Discussion

### Research gaps and robustness of data

Although the present study is based on a large number of invasive plant species worldwide, potential biases in the dataset may influence the robustness and generality of the results. The global coverage is rather heterogeneous in terms of regions, biomes and habitats (Table 2) and the majority of data come from temperate grasslands and woodlands of North America and Europe, which reflects well-known patterns in research intensity in biological invasions (Pyšek *et al*., 2008). Still, most of the combinations of environmental settings are covered by some data (Table 2). The 167 species included in this study represent 42% of invasive plant taxa for which published case studies on invasions exist (Pyšek *et al*., 2008) and 37% of the plants assessed were among the most serious environmental weeds worldwide (Weber, 2003). Although we are confident that we have covered much of the quantitative literature on impacts, these trends suggest that detailed knowledge on impacts remains unquantified for most alien plants (Vilà *et al*., 2009; Hulme, 2012). Furthermore, the frequency with which different species have been studied is strongly skewed, with 21 species accounting for 50.7% of all cases testing impacts on individual outcomes (Appendix S2 in Supporting Information). Nevertheless, our data set covers all major life forms, major biomes and geographical regions, and it is probably the best that can be extracted from available literature. It therefore adequately assesses the current state of knowledge but highlights that this knowledge base still requires improvement.

### Significance and directions of impact

Our study was based on statistical significance as assessed by the original studies. Although it can be argued that every additional new species incorporated into an ecosystem is likely to have an ecological impact of some sort, this is not necessarily true (Hejda & Pyšek, 2006; Meffin *et al*., 2010). Furthermore, even if true, by comparing multiple species and outcomes we are able to identify the outcomes most susceptible to change.

The impact of invasive species is often labelled as ‘negative’ or ‘positive’ but assessment brings about interpretation difficulties. For the effects on resident plants and animals, the interpretation is relatively straightforward; reduced values in population and community characteristics imply decreased vigour and population status. However, for soil characteristics, an increase in, for example, soil nutrients may not necessarily mean an improved state of the affected ecosystem. For example, in oligotrophic or early successional ecosystems increased nutrient status may lead to further invasion (Vitousek *et al*., 1987; Vitousek & Walker, 1989). It also needs to be noted that the direction of changes in pH has a different meaning than that in other soil characteristics, depending on the state before invasion. In the same vein, an increase in the frequency and/or intensity of fires, that is, the change in the natural fire regime, which often supports the invasive species (D'Antonio & Vitousek, 1992), represents rather undesirable change in ecosystem functioning. On the other hand, alien species may become components of novel ecosystems and may also help provide ecosystem services, new mutualistic relationships or increase the abundance of some native biota (Hobbs *et al*., 2006; Schweiger *et al*., 2010). Therefore, the ‘negative’ or ‘positive’ interpretation of the impact is a subjective assessment that has been used to rank alien species as non-desirable or desirable according to the interests of some economic sectors (Gozlan & Newton, 2009; but see Hulme *et al*., 2009a). The valid measure of impact is the net change compared to non-invaded (prior to invasion) situation independently of the direction of the change, and whether it can be labelled as ‘positive’ or ‘negative’ depends on human perception of that particular situation.

However, the direction of change provides important insights into how resident species, communities and ecosystems are affected by invading plants. Although the directions of impact could not be statistically tested in our study and therefore need to be interpreted with caution, several robust trends can be highlighted in terms of impact direction. Species and community outcomes tended to be reduced by plant invasions, which accords with previous studies that addressed mostly impacts on resident species richness and diversity (Vilà *et al*., 2006; Gaertner *et al*., 2009; Hejda *et al*., 2009), but these impacts were disproportionally more often significant on resident plants than animals. The abundance and richness of soil biota, similar to those of other soil measures, more often increased than decreased following invasion. Unfortunately, studies simultaneously investigating the impacts of alien plants on primary producers and on other trophic levels are scarce and only include a single species (Valtonen *et al*., 2006; de Groot *et al*., 2007; Gerber *et al*., 2008).

### Context dependence of impacts

Our analysis revealed that invasive plants exert consistent significant impacts on some outcomes, although for others the significance is context dependent and this is true whether impacts are examined at the species or community levels. Consistent outcomes include the survival of resident biota, activity of resident animals, resident community productivity, mineral and nutrient content in plant tissues and fire regime. However, there is a clear pattern in that for all the outcomes related to community richness and diversity, and to soil resources, the significance of impacts is determined by an interaction between invasive species traits and the biome invaded, regardless of the particular habitat and geographical region. These outcomes are most likely to be impacted if the invading plants fall into two broad groups: annual grasses (i.e. *Bromus tectorum* and several other *Bromus* spp., *Aegilops triuncialis*, *Microstegium vimineum*, *Avena barbata*, *Lolium multiflorum* and *Pennisetum polystachion*), and other life forms that are tall (the threshold of 4.8 m effectively means trees as indicated by this life form being a surrogate of height in the respective statistical model, with association value 0.53 corresponding to 40% of the improvement value of the primary splitter) and invade mediterranean or tropical regions (i.e. *Falcataria molluccana*, *Ailanthus altissima*, *Delairea odorata*, *Elaeagnus umbellata*, *Morella faya*, *Robinia pseudoacacia* and *Fraxinus uhdei*). In both groups, annual grasses and tall plants of other life forms invading the two biomes mentioned, impact is likely to be significant in more than 90% of cases. The general message from these findings is that (1) there is no universal measure of impact and so what we conclude depends on what we measure (Hulme, 2011, 2012), and (2) impact is also strongly context dependent (e.g. compare Hulme & Bremner, 2006 with Hejda & Pyšek, 2006 for contrasting impacts of *Impatiens glandulifera*; see also Thiele *et al*., 2010b).

### Impacts on resident species richness and productivity: prone to prediction?

One of the clearest signals in this analysis is that invasive plants are far more likely to cause significant impacts on resident plant and animal richness on islands rather than mainlands. In terms of plant invaders, this result seems to be generally valid since among them there are plant species of various life forms represented and their invasions occurred in multiple biomes and regions. However, it needs to be noted that although the islands included in our study represent all continents, they are limited in numbers to those in which impact was studied (Seychelles, Prince Edwards Island, Sri Lanka, coast of China, New Zealand, Mediterranean islands, United Kingdom, Hawaii, coast of Patagonia; see Appendix S1 in Supporting Information) and do not represent a random sample of islands worldwide. Data were insufficient to disentangle whether or not the island effect could be attributed primarily to isolated oceanic rather than continental islands, nor could islands be paired with their nearest representative mainland regions (e.g. Gimeno *et al*., 2006). However, the sample does include some of the most invaded island ecosystems in the world, for example, Hawaii or New Zealand and these may not be representative of islands worldwide.

The pattern on mainland was, again, context dependent; animal species richness was less likely to be impacted by plant invasions, which is consistent with impact diminishing at higher trophic levels that might reflect bottom-up control of impacts (de Groot *et al*., 2007; Scherber *et al*., 2010). Furthermore, on mainlands invasive plants pollinated by wind encompassing a wide range of life forms, statures and taxonomic affiliations (e.g. *Agropyron cristatum*, *Bromus tectorum*, *Carex kobomugi*, *Carpinus betulus*, *Cortaderia selloana*, *Juniperus pinchotii*, *Pinus contorta* or *Rumex alpinus*) were highly likely to exert significant impacts on plant species richness. One explanation of this result could be that the fecundity of wind pollinated species will not be dependent on the availability of pollinators and this allows them to build a high local cover resulting in significant impact on plant community diversity. However, it also is certainly possible that wind pollination may be correlated with some unmeasured trait that leads to the impacts observed. Alternatively, given the rather diverse group of plant species that comprised this splitter, other plant species traits and site characteristics on mainland have relatively small effects on species richness. Nevertheless, this still suggests that knowledge of wind pollination may be a useful proxy for such unmeasured traits. Consistent with this general finding regarding wind pollination, invasive plants of low stature (<1.2 m) requiring pollinators generally did not cause significant impact on plant species richness. However, this appears to reflect the height of species that usually dominate the herb layer in many plant communities of most biomes analysed rather than pollination syndrome (Ellenberg, 1988; Hejda *et al*., 2009; Thiele *et al*., 2010a).

An indication of the direction of these effects can be inferred from the fact that the majority of significant impacts resulted in decreased species richness and diversity (Fig. 1). For islands, only one case study (*Tradescantia fluminensis* invasion in New Zealand) revealed an increase in resident species richness (but this was in the seed bank), whereas richness of seedlings was reduced (Standish *et al*., 2001). This overall trend is supported by the trends observed in mediterranean-type ecosystems worldwide where a significant negative effect of invasions on resident species richness was found, with the strength of this effect depending on the life form of the invading plant, invaded habitat and the scale and character of the data (Gaertner *et al*., 2009).

Similarly consistent patterns were also found for impacts on productivity, with invasive plant height clearly discriminating the likelihood of significance – plants taller than 1.2 m, that is, the usual dominants of the herb layer (Ellenberg, 1988), were more likely to exert a significant impact than shorter plants. Finally, our results indicate that the significance of the impact of a plant invasion on soil resources cannot be predicted based on species traits or environmental context, it rather depends on the identity of individual species and their taxonomic affiliation (at the level of genus). This probably again reflects that measures of impact addressing indirect relationships with trophic levels involved in nutrient cycling are more difficult to predict because they are largely driven by species-specific plant–soil relationships (Inderjit & van der Putten, 2010). It seems also plausible that site history and location are more important for ecosystem consequences of invasion, especially for elements such as P and cations which are less mobile than C and N (Ehrenfeld, 2010).

### Towards risk analysis that assesses consequences of plant invasions?

Although the significance of impacts was context dependent, this context only rarely included environmental variables such as site characteristics. For example, only insularity and mediterranean biome were found to be important determinants of impact on species richness whereas habitat and region were never significant variables in any analyses. Furthermore, taxonomic affiliation of the species had no detectable effect on the probability of it exerting a significant impact. Thus, generalizations on impact based on higher taxonomic levels can be misleading, and assessments should be primarily made at a species level, similarly to assessments of the risk of invasion (Pyšek *et al*., 2009, 2011; Moravcová *et al*., 2010).

There was a much stronger signal that species traits may provide a means to predict impact, especially life form, stature and pollination syndrome. The question whether or not the same traits confer both invasiveness as well as significant impact, although important, has rarely been addressed, if at all (Levine *et al*., 2003). Our study indicates that, depending on context, some of the traits conferring invasiveness, namely life form and height of the invader correspond to traits regarded as potentially influencing invasion success (Pyšek & Richardson, 2007). However, due to the constraints resulting from the different nature of studies examining invasion success (Pyšek *et al*., 2009, 2010), the compatibility of traits conferring invasiveness and impact cannot yet be assessed and research that would specifically address correspondence between the two consequences of plant introduction is much needed (Hulme, 2011, 2012). If both invasiveness and impact are associated with a similar suite of traits, the body of information available from screening systems addressing invasiveness (e.g. Pheloung *et al*., 1999; Daehler & Carino, 2000; Daehler *et al*., 2004; Weber & Gut, 2004; Gordon *et al*., 2008) would be also applicable to impact which is, from the management point of view, a more important measure (Hulme, 2006).

## Supporting Information

**Appendix S1.** References to case studies on impact of invasive plants.

**Appendix S2.** List of species that appeared in studies on impact of invasive plants included in the study. Please note: Wiley-Blackwell are not responsible for the content or functionality of any supporting materials supplied by the authors. Any queries (other than missing material) should be directed to the corresponding author for the article.
