# Supplementary material for: A global assessment of invasive plant impacts on resident species, communities and ecosystems: the interaction of impact measures, invading species' traits and environment
Source: Glob Chang Biol. 2012 May;18(5):1725–37. doi: 10.1111/j.1365-2486.2011.02636.x (PMC3597245; doi:10.1111/j.1365-2486.2011.02636.x)
Supplement: Supplementary file 1 — Appendix S1. References to case studies on impact of invasive plants. [file gcb0018-1725-SD1.doc]

**Appendix S1.** References to case studies on impact of invasive plants reviewed in the present paper.

Able, K.W. & Ragan, S.M. (2003) Impact of common reed, *Phragmites australis*, on essential fish habitat: Influence on reproduction, embryological development, and larval abundance of mummichog (*Fundulus heteroclitus*). *Estuaries*, **26**, 40–50.

Allison, S.D., Nielsen, C. & Hughes, R.F. (2006) Elevated enzyme activities in soils under the invasive nitrogen-fixing tree *Falcataria moluccana*. *Soil Biology & Biochemistry*, **38**, 1537–1544.

Alvarez, M.E. & Cushman, J.H. (2002) Community-level consequences of a plant invasion: effects on three habitats in coastal California. *Ecological Applications*, **12**, 1434–1444.

Amiotti, N.M., Zalba, P., Sanchez, L.F. & Peinemann, N. (2000) The impact of single trees on properties of loess-derived grassland soils in Argentina. *Ecology*, **81**, 3283–3290.

Angeloni, N.L., Jankowski, K.J., Tuchman, N.C. & Kelly, J.J. (2006) Effects of an invasive cattail species (*Typha* x *glauca*) on sediment nitrogen and microbial community composition in a freshwater wetland. *Fems Microbiology Letters*, **263**, 86–92.

Ashton, P.M.S., Gamage, S., Gunatilleke, I. & Gunatilleke, C.V.S. (1997) Restoration of a Sri Lankan rainforest: using Caribbean pine *Pinus caribaea* as a nurse for establishing late-successional tree species. *Journal of Applied Ecology*, **34**, 915–925.

Asner, G.P. & Beatty, S.W. (1996) Effects of an African grass invasion on Hawaiian shrubland nitrogen biogeochemistry. *Plant and Soil*, **186**, 205–211.

Asner, G.P. & Vitousek, P.M. (2005) Remote analysis of biological invasion and biogeochemical change. *Proceedings of the National Academy of Sciences of the United States of America*, **102**, 4383–4386.

Asner, G.P., Hughes, R.F., Vitousek, P.M., Knapp, D.E., Kennedy-Bowdoin, T., Boardman, J., Martin, R.E., Eastwood, M. & Green, R.O. (2008) Invasive plants transform the three-dimensional structure of rain forests. *Proceedings of the National Academy of Sciences of the United States of America*, **105**, 4519–4523.

Auerbach, M. & Simberloff, D. (1988) Rapid leaf-miner colonization of introduced trees and shifts in sources of herbivore mortality. *Oikos*, **52**, 41–50.

Badano, E.I. & Pugnaire, F.I. (2004) Invasion of *Agave* species (Agavaceae) in south-east Spain: invader demographic parameters and impacts on native species. *Diversity and Distributions*, **10**, 493–500.

Badano, E.I., Jones, C.G., Cavieres, L.A. & Wright, J.P. (2006) Assessing impacts of ecosystem engineers on community organization: a general approach illustrated by effects of a high-Andean cushion plant. *Oikos*, **115**, 369–385.

Baer, S.G., Church, J.M., Williard, K.W.J. & Groninger, J.W. (2006) Changes in intrasystem N cycling from N-2-fixing shrub encroachment in grassland: multiple positive feedbacks. *Agriculture Ecosystems & Environment*, **115**, 174–182.

Bakker, J. & Wilson, S. (2001) Competitive abilities of introduced and native grasses. *Plant Ecology*, **157**, 117–125.

Barclay, A.D., Betancourt, J.L. & Allen, C.D. (2004) Effects of seeding ryegrass (*Lolium multiflorum*) on vegetation recovery following fire in ponderosa pine (*Pinus ponderosa*) forest. *International Journal of Wildland Fire*, **13**, 183–194.

Bartomeus, I., Vilà, M. & Santamaria, L. (2008) Contrasting effects of invasive plants in plant-pollinator networks. *Oecologia*, **155**, 761–770.

Batten, K.M., Scow, K.M. & Espeland, E.K. (2008) Soil microbial community associated with an invasive grass differentially impacts native plant performance. *Microbial Ecology*, **55**, 220–228.

Beatley, J.C. (1966) Ecological status of introduced brome grasses (*Bromus* spp.) in desert vegetation of Southern Nevada. *Ecology*, **47**, 548–554.

Bell, G.P. (1997) Ecology and management of *Arundo donax*, and approaches to riparian habitat restoration in Southern California. *Plant invasions: studies from North America and Europe* (ed. J. H. Brook), pp. 103–113, Backhuys Publishers, Leiden.

Belnap, J. & Phillips, S.L. (2001) Soil biota in an ungrazed grassland: response to annual grass (*Bromus tectorum*) invasion. *Ecological Applications*, **11**, 1261–1275.

Belnap, J., Phillips, S.L. & Troxler, T. (2006) Soil lichen and moss cover and species richness can be highly dynamic: the effects of invasion by the annual exotic grass *Bromus tectorum*, precipitation, and temperature on biological soil crusts in SE Utah. Applied Soil Ecology, *Biological Invasions and Belowground Ecology*, **32**, 63–76.

Belnap, J., Phillips, S.L., Sherrod, S.K. & Moldenke, A. (2005) Soil biota can change after exotic plant invasion: does this affect ecosystem processes? *Ecology*, **86**, 3007–3017.

Biggerstaff, M.S. & Beck, C.W. (2007) Effects of English ivy (*Hedera helix*) on seed bank formation and germination. *American Midland Naturalist*, **157**, 250–257.

Blank, R.R. & Young, J.A. (2002) Influence of the exotic invasive crucifer, *Lepidium latifolium*, on soil properties and elemental cycling. *Soil Science*, **167**, 821–829.

Blank, R.R. (2008) Biogeochemistry of plant invasion: a case study with downy brome (*Bromus tectorum*). *Invasive Plant Science and Management*, **1**, 226–238.

Bock, C.E., Kennedy, L., Bock, J.H. & Jones, Z.F. (2007 Effects of fire frequency and intensity on velvet mesquite in an Arizonian grassland. *Rangeland Ecology and Management*, **60**, 508–514.

Booth, M.S., Stark, J.M. & Caldwell, M.M. (2003) Inorganic N turnover and availability in annual- and perennial-dominated soils in a Northern Utah shrub-steppe ecosystem. *Biogeochemistry*, **66**, 311–330.

Borgmann, K.L. & Rodewald, A.D. (2004) Nest predation in an urbanizing landscape: the role of exotic shrubs. *Ecological Applications*, **14**, 1757–1765.

Boswell, C.C. & Espie, P.R. (1998) Uptake of moisture and nutrients by *Hieracium pilosella* and effects on soil in a dry sub-humid grassland. *New Zealand Journal of Agricultural Research*, **41**, 251–261.

Bowers, M.D., Stamp, N.E. & Collinge, S.K. (1992) Early stage of host range expansion by a specialist herbivore, *Euphydryas-Phaeton* (Nymphalidae). *Ecology*, **73**, 526–536.

Bradley, B.A., Houghton, R.A., Mustard, J.F. & Hamburg, S.P. (2006) Invasive grass reduces aboveground carbon stocks in shrublands of the Western US. *Global Change Biology*, **12**, 1815–1822.

Braithwaite, R.W., Lonsdale, W.M. & Estbergs, J.A. (1989) Alien vegetation and native biota in tropical Australia: the impact of *Mimosa pigra*. *Biological Conservation*, **48**, 189–210.

Brandon, A.L., Gibson, D.J. & Middleton, B.A. (2004) Mechanisms for dominance in an early successional old field by the invasive non-native *Lespedeza cuneata* (Dum. Cours.) G. Don. *Biological Invasions*, **6**, 483–493.

Bray, S.R., Kitajima, K. & Sylvia, D.M. (2003) Mycorrhizae differentially alter growth, physiology, and competitive ability of an invasive shrub. *Ecological Applications*, **13**, 565–574.

Brooks, M.L. (2000) Competition between alien annual grasses and native annual plants in the Mojave Desert. *American Midland Naturalist*, **144**, 92–108.

Brown, B.J. & Mitchell, R.J. (2001) Competition for pollination: effects of pollen of an invasive plant on seed set of a native congener. *Oecologia*, **129**, 43–49.

Brown, C.J., Blossey, B., Maerz, J.C. & Joule, S.J. (2006) Invasive plant and experimental venue affect tadpole performance. *Biological Invasions*, **8**, 327–338.

Brown, D.E. & Minnich, R.A. (1986) Fire and changes in creosote bush scrub of the western Sonoran Desert, California. *American Midland Naturalist*, **116**, 411–422.

Busch, D.E. & Smith, S.D. (1995) Mechanisms associated with decline of woody species in riparian ecosystems of the Southwestern US. *Ecological Monographs*, **65**, 347–370.

Butler, D.W. & Fairfax, R.J. (2003) Buffel grass and fire in a Gidgee and Brigalow woodland: a case study from central Queensland. *Ecological Management & Restoration*, **4**, 120–125.

Cable, D.R. (1971) Lehmann lovegrass on the Santa Rita Experimental Range, 1937–1968 (El Zacate Lehmann lovegrass en la Estacion Experimental de Santa Rita Durante los anos 1937–68). *Journal of Range Management*, **24**, 17–21.

Caldwell, B.A. (2006) Effects of invasive scotch broom on soil properties in a Pacific coastal prairie soil. *Applied Soil Ecology*, **32**, 149–152.

Cameron, G.N. & Spencer, S.R. (1989) Rapid leaf decay and nutrient release in a Chinese tallow forest. *Oecologia*, **80**, 222–228.

Cannon, J.P., Allen, E.B., Allen, M.F., Dudley, L.M. & Jurinak, J.J. (1995) The effects of oxalates produced by *Salsola tragus* on the phosphorus-nutrition of *Stipa pulchra* . *Oecologia*, **102**, 265–272.

Carroll, S.P., Klassen, S.T.P. & Dingle, H. (1998) Rapidly evolving adaptations to host ecology and nutrition in the soapberry bug. *Evolutionary Ecology*, **12**, 955–968.

Castro-Díez, P., González-Muńoz, N., Alonso, A., Gallardo, A. & Poorter, L. (2009) Effects of exotic invasive trees on nitrogen cycling: a case study in Central Spain. *Biological Conservation*, **11**, 1973–1986.

Chapuis-Lardy, L., Vanderhoeven, S., Dassonville, N., Koutika, L.S. & Meerts, P. (2006) Effect of the exotic invasive plant *Solidago gigantea* on soil phosphorus status. *Biology and Fertility of Soils*, **42**, 481–489.

Chen, B.M., Peng, S.L. & Ni, G.Y. (2009) Effects of the invasive plant *Mikania micrantha* H.B.K. on soil nitrogen availability through allelopathy in South China. *Biological Invasions*, **11**, 1291–1299.

Chen, H., Li, B., Fang, C., Chen, J. & Wu, J. (2007) Exotic plant influences soil nematode communities through litter input. *Soil Biology & Biochemistry*, **39**, 1782–1793.

Chen, Z.Y., Li, B., Zhong, Y. & Chen, J.K. (2004) Local competitive effects of introduced *Spartina alterniflora* on *Scirpus mariqueter* at Dongtan of Chongming Island, the Yangtze River estuary and their potential ecological consequences. *Hydrobiologia*, **528**, 99–106.

Chittka, L. & Schurkens, S. (2001) Successful invasion of a floral market: an exotic Asian plant has moved in on Europe's river-banks by bribing pollinators. *Nature*, **411**, 653–653.

Christian, J.M. & Wilson, S.D. (1999) Long-term ecosystem impacts of an introduced grass in the northern Great Plains. *Ecology*, **80**, 2397–2407.

Cipollini, K.A., McClain, G.Y. & Cipollini, D. (2008) Separating above- and belowground effects of *Alliaria petiolata* and *Lonicera maackii* on the performance of *Impatiens capensis*. *American Midland Naturalist*, **160**, 117–128.

Cline, J.F., Uresk, D.W. & Rickard, W.H. (1977) Comparison of soil-water used by a sagebrush-bunchgrass and a cheatgrass community. *Journal of Range Management*, **30**, 199–201.

Collier, M.H., Vankat, J.L. & Hughes, M.R. (2002) Diminished plant richness and abundance below *Lonicera maackii*, an invasive shrub. *American Midland Naturalist*, **147**, 60–71.

Cordell, S. & Sandquist, D.R. (2008) The impact of an invasive African bunchgrass (*Pennisetum setaceum*) on water availability and productivity of canopy trees within a tropical dry forest in Hawaii. *Functional Ecology*, **22**, 1008–1017.

Cronin, J.T. & Haynes, K.J. (2004) An invasive plant promotes unstable host-para-sitoid patch dynamics. *Ecology*, **85**, 2772–2782.

Crooks, J.A. (2002) Characterizing ecosystem-level consequences of biological invasions: the role of ecosystem engineers. *Oikos*, **97**, 153–166.

Daehler, C.C. & Carino, D.A. (1998) Recent replacement of native pili grass (*Heteropogon contortus*) by invasive African grasses in the Hawaiian islands. *Pacific Science*, **52**, 220–227.

D'Antonio, C.M. & Mahall, B.E. (1991) Root profiles and competition between the invasive, exotic perennial, *Carpobrotus edulis*, and 2 native shrub species in California coastal scrub. American *Journal of Botany*, **78**, 885–894.

D'Antonio, C.M., Hughes, R.F., Mack, M., Hitchcock, D. & Vitousek, P.M. (1998) The response of native species to removal of invasive exotic grasses in a seasonally dry Hawaiian woodland. *Journal of Vegetation Science*, **9**, 699–712.

D'Antonio, C.M. & Vitousek, P.M. (1992) Biological invasions by exotic grasses, the grass/fire cycle, and global change. *Annual Review of Ecological Systems*, **23**, 63–87.

Dascanio, L.M., Barrera, M.D. & Frangi, J.L. (1994) Biomass structure and dry-matter dynamics of subtropical alluvial and exotic *Ligustrum* forests at The Rio de La Plata, Argentina. *Vegetatio*, **115**, 61–76.

Dassonville, N., Vanderhoeven, S., Gruber, W. & Meerts, P. (2007) Invasion by *Fallopia japonica* increases topsoil mineral nutrient concentrations. *Ecoscience*, **14**, 230–240.

Davalos, A. & Blossey, B. (2004) Influence of the invasive herb garlic mustard (*Alliaria petiolata*) on ground beetle (Coleoptera: Carabidae) assemblages. *Environmental Entomology*, **33**, 564–576.

de Groot, M., Kleijn, D. & Jogan, N. (2007) Species groups occupying different trophic levels respond differently to the invasion of semi-natural vegetation by *Solidago canadensis*. *Biological Conservation*, **136**, 612–617.

De Rouw, A. (1991) The invasion of *Chromolaena odorata* (L.) King & Robinson (ex *Eupatorium odoratum*), and the competition with the native flora, in a rain forest zone, south-west Cote d'Ivoire. *Journal of Biogeography*, **18**, 13–23.

Dean, W.R.J., Anderson, M.D., Milton, S.J. & Anderson, T.A. (2002) Avian assemblages in native *Acacia* and alien *Prosopi*s drainage line woodland in the Kalahari, South Africa. *Journal of Arid Environments*, **51**, 1–19.

DeFalco, L.A., Fernandez, G.C.J. & Nowak, R.S. (2007) Variation in the establishment of a non-native annual grass influences competitive interactions with Mojave Desert perennials. *Biological Invasions*, **9**, 293–307.

Denoth, M. & Myers, J.H. (2007) Competition between *Lythrum salicaria* and a rare species: combining evidence from experiments and long-term monitoring. *Plant Ecology*, **191**, 153–161.

Dillemuth, F.P., Rietschier, E.A. & Cronin, J.T. (2009) Patch dynamics of a native grass in relation to the spread of invasive smooth brome (*Bromus inermis*). *Biological Invasions*, **11**, 1381–1391.

Dillenburg, L.R., Whigham, D.F., Teramura, A.H. & Forseth, I.N. (1993) Effects of belowground and aboveground competition from the vines *Lonicera japonica* and *Parthenocissus quinquefolia* on the growth of the tree host *Liquidambar styraciflua*. *Oecologia*, **93**, 48–54.

Domenech, R. & Vilà, M. (2008) Response of the invader *Cortaderia selloana* and two coexisting natives to competition and water stress. *Biological Invasions*, **10**, 903–912.

Domenech, R., Vilà, M., Gesti, J. & Serrasolses, I. (2006) Neighbourhood association of *Cortaderia selloana* invasion, soil properties and plant community structure in Mediterranean coastal grasslands. *Acta Oecologica-International Journal of Ecology*, **29**, 171–177.

Dormaar, J.F., Naeth, M.A., Willms, W.D. & Chanasyk, D.S. (1995) Effect of native prairie, crested wheatgrass (*Agropyron cristatum* (L.) Gaertn.) and Russian wildrye (*Elymus junceus* Fisch.) on soil chemical-properties. *Journal of Range Management*, **48**, 258–263.

Douglas, M.M. & O'Connor, R.A. (2003) Effects of the exotic macrophyte, para grass (*Urochloa mutica*), on benthic and epiphytic macroinvertebrates of a tropical floodplain. *Freshwater Biology*, **48**, 962–971.

Douglas, M.M., Setterfield, S.A., Rossiter, N., Barratt, J. & Hutley, L.B. (2004) Effects of mission grass (*Pennisetum polystachion* (L.) Schult.) invasion on fuel loads and nitrogen availability in a northern Australia tropical savanna. *Weed management: balancing people, planet, profit. Fourteenth Australian Weeds Conference* (eds B. M. [Sindel &](http://www.cabdirect.org:80/search.html?q=ed%3A"Sindel%2C+B.+M.")  S. B. [Johnson)](http://www.cabdirect.org:80/search.html?q=ed%3A"Johnson%2C+S.+B."), pp. 179–181. Wagga Wagga, New South Wales, Australia.

Drenovsky, R.E. & Batten, K.M. (2007) Invasion by *Aegilops triuncialis* (barb goatgrass) slows carbon and nutrient cycling in a serpentine grassland. *Biological Invasions*, **9**, 107–116.

Dunbar, K.R. & Facelli, J.M. (1999) The impact of a novel invasive species, *Orbea variegata* (African carrion flower), on the chenopod shrublands of South Australia. *Journal of Arid Environments*, **41**, 37–48.

Durst, S.L., Theimer, T.C., Paxton, E.H. & Sogge, M.K. (2008) Temporal variation in the arthropod community of desert riparian habitats with varying amounts of saltcedar (*Tamarix ramosissima*). *Journal of Arid Environments*, **72**, 1644–1653.

Egunjobi, J.K. (1969) Dry matter and nitrogen accumulation in secondary successions involving gorse (*Ulex europaeus* L.) and associated shrubs and trees. *New Zealand Journal of Science*, **12**, 175–193.

Ehrenfeld, J.G., Kourtev, P. & Huang, W.Z. (2001) Changes in soil functions following invasions of exotic understory plants in deciduous forests. *Ecological Applications*, **11**, 1287–1300.

El-Ghareeb, R. (1991) Vegetation and soil changes induced by *Mesembranthemum crystallinum* L. in a Mediterranean desert ecosystem. *Journal of Arid Environment*, **20**, 321–330.

Ellingson, A.R. & Andersen, D.C. (2002) Spatial correlations of *Diceroprocta apache* and its host plants: evidence for a negative impact from *Tamarix* invasion. *Ecological Entomology*, **27**, 16–24.

Ellis, L.M., Crawford, C.S. & Molles, M.C. (1998) Comparison of litter dynamics in native and exotic riparian vegetation along the Middle Rio Grande of central New Mexico, USA. *Journal of Arid Environments*, **38**, 283–296.

Enloe, S.F., DiTomaso, J.M., Orloff, S.B. & Drake, D.J. (2004) Soil water dynamics differ among rangeland plant communities dominated by yellow starthistle (*Centaurea solstitialis*), annual grasses, or perennial grasses. *Weed Science*, **52**, 929–935.

Ens, E.J. & French, K. (2008) Exotic woody invader limits the recruitment of three indigenous plant species. *Biological Conservation*, **141**, 590–595.

Ernst, C.M. & Cappuccino, N. (2005) The effect of an invasive alien vine, *Vincetoxicum rossicum* (Asclepiadaceae), on arthropod populations in Ontario old fields. *Biological Invasions*, **7**, 417–425.

Evans, R.D., Rimer, R., Sperry, L. & Belnap, J. (2001) Exotic plant invasion alters nitrogen dynamics in an arid grassland. *Ecological Applications*, **11**, 1301–1310.

Farnsworth, E.J. & Meyerson, L.A. (1999) Species composition and inter-annual dynamics of a freshwater tidal plant community following removal of the invasive grass, *Phragmites australis*. *Biological Invasions*, **1**, 115–127.

Feare, C.J., Gill, E.L., Carty, P., Carty, H.E. & Ayrton, V.J. (1997) Habitat use by Seychelles sooty terns *Sterna fuscata* and implications for colony management. *Biological Conservation*, **81**, 69–76.

Fickbohm, S.S. & Zhu, W.-X. (2006) Exotic purple loosestrife invasion of native cattail freshwater wetlands: effects on organic matter distribution and soil nitrogen cycling. *Applied Soil Ecology*, **32**, 123–131.

Finch, O.D. & Szumelda, A. (2007) Introduction of Douglas fir (*Pseudotsuga menziesii* (Mirb.) Franco) into Western Europe: epigaeic arthropods in intermediate-aged pure stands in northwestern Germany. *Forest Ecology and Management*, **242**, 260–272.

Fisher, J.L., Veneklaas, E.J., Lambers, H. & Loneragan, W.A. (2006) Enhanced soil and leaf nutrient status of a Western Australian *Banksia* woodland community invaded by *Ehrharta calycina* and *Pelargonium capitatum*. *Plant and Soil*, **284**, 253–264.

Fleishman, E., Mac Nally, R. & Murphy, D.D. (2005) Relationships among non-native plants, diversity of plants and butterflies, and adequacy of spatial sampling. *Biological Journal of the Linnean Society*, **85**, 157–166.

Frappier, B., Eckert, R.T. & Lee, T.D. (2003) Potential impacts of the invasive exotic shrub *Rhamnus frangula* L. (glossy buckthorn) on forests of southern New Hampshire. *Northeastern Naturalist*, **10**, 277–296.

Freifelder, R.R., Vitousek, P.M. & D'Antonio, C.M. (1998) Microclimate change and effect on fire following forest-grass conversion in seasonally dry tropical woodland. *Biotropica*, **30**, 286–297.

French, K. & Major, R.E. (2001) Effect of an exotic *Acacia* (Fabaceae) on ant assemblages in South African fynbos. *Austral Ecology*, **26**, 303–310.

French, K. & Zubovic, A. (1997) Effect of the weed *Chrysanthemoides monilifera* (bitou bush) on bird communities. *Wildlife Research*, **24**, 727–735.

Funk, J.L. (2005) *Hedychium gardnerianum* invasion into Hawaiian montane rainforest: interactions among litter quality, decomposition rate, and soil nitrogen availability. *Biogeochemistry*, **76**, 441–451.

Galbraith-Kent, S.L. & Handel, S.N. (2008) Invasive *Acer platanoides* inhibits native sapling growth in forest understorey communities. *Journal of Ecology*, **96**, 293–302.

Gentle, C.B. & Duggin, J.A. (1997) Allelopathy as a competitive strategy in persistent thickets of *Lantana camara* L. in three Australian forest communities. *Plant Ecology*, **132**, 85–95.

Gentle, C.B. & Duggin, J.A. (1998) Interference of *Choricarpia leptopetala* by *Lantana camara* with nutrient enrichment in mesic forests on the central coast of NSW. *Plant Ecology*, **136**, 205–211.

Ghazoul, J. (2004) Alien abduction: disruption of native plant-pollinator interactions by invasive species. *Biotropica*, **36**, 156–164.

Giantomasi, A., Tecco, P.A., Funes, G., Gurvich, D.E. & Cabido, M. (2008) Canopy effects of the invasive shrub *Pyracantha angustifolia* on seed bank composition, richness and density in a montane shrubland (Cordoba, Argentina). *Austral Ecology*, **33**, 68–77.

Goergen, E. & Daehler, C.C. (2001) Inflorescence damage by insects and fungi in native pili grass ( *Heteropogon contortus* ) versus alien foundation grass (*Pennisetum setaceum* ) in Hawai'i. *Pacific Science*, **55**, 129–136.

Going, B.M. & Dudley, T.L. (2008) Invasive riparian plant litter alters aquatic insect growth. *Biological Invasions*, **10**, 1041–1051.

Gomez-Aparicio, L. & Canham, C.D. (2008) Neighbourhood analyses of the allelopathic effects of the invasive tree *Ailanthus altissima* in temperate forests. *Journal of Ecology*, **96**, 447–458.

Gomez-Gonzalez, S. & Cavieres, L.A. (2009) Litter burning does not equally affect seedling emergence of native and alien species of the Mediterranean-type Chilean matorral. *International Journal of Wildland Fire*, **18**, 213–221.

Gordon, D.R. (1998) Effects of invasive, non-indigenous plant species on ecosystem processes: lessons from Florida. *Ecological Applications*, **8**, 975–989.

Gould, A.M.A. & Gorchov, D.L. (2000) Effects of the exotic invasive shrub *Lonicera maackii* on the survival and fecundity of three species of native annuals. *American Midland Naturalist*, **144**, 36–50.

Gratton, C. & Denno, R.F. (2006) Arthropod food web restoration following removal of an invasive wetland plant. *Ecological Applications*, **16**, 622–631.

Greenwood, H., O'Dowd, D.J. & Lake, P.S. (2004) Willow (*Salix* x *rubens*) invasion of the riparian zone in south-eastern Australia: reduced abundance and altered composition of terrestrial arthropods. *Diversity and Distributions*, **10**, 485–492.

Gremmen, N.J.M., Chown, S.L. & Marshall, D.J. (1998) Impact of the introduced grass *Agrostis stolonifera* on vegetation and soil fauna communities at Marion Island, sub-Antarctic. *Biological Conservation*, **85**, 223–231.

Guerrero, P.C. & Bustamante, R.O. (2007) Can native tree species regenerate in *Pinus radiata* plantations in Chile? Evidence from field and laboratory experiments. *Forest Ecology and Management*, **253**, 97–102.

Hacker, S.D. & Dethier, M.N. (2006) Community modification by a grass invader has differing impacts for marine habitats. *Oikos*, **113**, 279–286.

Hager, H.A. (2004) Competitive effect versus competitive response of invasive and native wetland plant species. *Oecologia*, **139**, 140–149.

Haidinger, T.L. & Keeley, J.E. (1993) Role of high fire frequency in destruction of mixed chaparral. *Madrono*, **40**, 141–147.

Hall, S.J. & Asner, G.P. ( 2007) Biological invasion alters regional nitrogen-oxide emissions from tropical rainforests. *Global Change Biology*, **13**, 2143–2160.

Harcombe, P.A., Cameron, G.N. & Glumac, E.G. (1993) Aboveground net primary productivity in adjacent grassland and woodland on the coastal prairie of Texas, USA. *Journal of Vegetation Science*, **4**, 521–530.

Hartman, K.M. & McCarthy, B.C. (2004) Restoration of a forest understory after the removal of an invasive shrub, Amur honeysuckle (*Lonicera maackii*). *Restoration Ecology*, **12**, 154–165.

Hartman, K.M. & McCarthy, B.C. (2007) A dendro-ecological study of forest overstorey productivity following the invasion of the non-indigenous shrub *Lonicera maackii*. *Applied Vegetation Science*, **10**, 3–14.

Haubensak, K.A. & Parker, I.M. (2004) Soil changes accompanying invasion of the exotic shrub *Cytisus scoparius* in glacial outwash prairies of western Washington, USA. *Plant Ecology*, **175**, 71–79.

Hawkes, C.V., Belnap, J., D'Antonio, C. & Firestone, M.K. (2006) Arbuscular mycorrhizal assemblages in native plant roots change in the presence of invasive exotic grasses. *Plant and Soil*, **281**, 369–380.

Hedge, P. & Kriwoken, L.K. (2000) Evidence for effects of *Spartina anglica* invasion on benthic macrofauna in Little Swanport estuary, Tasmania. *Austral Ecology*, **25**, 150–159.

Hejda, M. & Pyšek, P. (2006) What is the impact of *Impatiens glandulifera* on species diversity of invaded riparian vegetation? *Biological Conservation*, **132**, 143–152.

Hejda, M., Pyšek, P. & Jarošík, V. (2009) Impact of invasive plants on the species richness, diversity and composition of invaded communities. *Journal of Ecology*, **97**, 393–403.

Henderson, D.C. & Naeth, M.A. (2005) Multi-scale impacts of crested wheatgrass invasion in mixed-grass prairie. *Biological Invasions*, **7**, 639–650.

Heneghan, L., Fatemi, F., Umek, L., Grady, K., Fagen, K. & Workman, M. (2006) The invasive shrub European buckthorn (*Rhamnus cathartica* L.) alters soil properties in Midwestern US woodlands. *Applied Soil Ecology*, **32**, 142–148.

Herrera, A.M. & Dudley, T.L. (2003) Reduction of riparian arthropod abundance and diversity as a consequence of giant reed (*Arundo donax*) invasion. *Biological Invasions*, **5**, 167–177.

Hoffmann, W.A. & Haridasan, M. (2008) The invasive grass, *Melinis minutiflora*, inhibits tree regeneration in a Neotropical savanna. *Austral Ecology*, **33**, 29–36.

Hoffmann, W.A., Lucatelli, V., Silva, F.J., Azeuedo, I.N.C., Marinho, M.D., Albuquerque, A.M.S., Lopes, A.D. & Moreira, S.P. (2004) Impact of the invasive alien grass *Melinis minutiflora* at the savanna-forest ecotone in the Brazilian Cerrado. *Diversity and Distributions*, **10**, 99–103.

Holly, D.C., Ervin, G.N., Jackson, C.R., Diehl, S.V. & Kirker, G.T. (2009) Effect of an invasive grass on ambient rates of decomposition and microbial community structure: a search for causality. *Biological Invasions*, **11**, 1855–1868.

Holmes, P.M. & Cowling, R.M. (1997) The effects of invasion by *Acacia saligna* on the guild structure and regeneration capabilities of South African fynbos shrublands. *Journal of Applied Ecology*, **34**, 317–332.

Hook, P.B., Olson, B.E. & Wraith, J.M. (2004) Effects of the invasive forb *Centaurea maculosa* on grassland carbon and nitrogen pools in Montana, USA. *Ecosystems*, **7**, 686–694.

Hoopes, M.F. & Hall, L.M. (2002) Edaphic factors and competition affect pattern formation and invasion in a California grassland. *Ecological Applications*, **12**, 24–39.

Houston, W.A. & Duivenvoorden, L.J. (2002) Replacement of littoral native vegetation with the ponded pasture grass *Hymenachne amplexicaulis*: effects on plant, macroinvertebrate and fish biodiversity of backwaters in the Fitzroy River, Central Queensland, Australia. *Marine and Freshwater Research*, **53**, 1235–1244.

Huenneke, L.F. & Thomson, J.K. (1995) Potential interference between a threatened endemic thistle and an invasive nonnative plant. *Conservation Biology*, **9**, 416–425.

Hughes, R.F. & Denslow, J.S. (2005) Invasion by a N-2-fixing tree alters function and structure in wet lowland forests of Hawaii. Ecological Applications, 15, 1615–1628.

Hughes, R.F. & Uowolo, A. (2006) Impacts of *Falcataria moluccana* invasion on decomposition in Hawaiian lowland wet forests: the importance of stand-level controls. *Ecosystems*, **9**, 977–991.

Hughes, F., Vitousek, P.M. & Tunison, T. (1991) Alien grass invasion and fire in the seasonal submontane zone of Hawaii. *Ecology*, **72**, 743–746.

Hulme, P.E. & Bremner, E.T. (2006) Assessing the impact of *Impatiens glandulifera* on riparian habitats: partitioning diversity components following species removal. *Journal of Applied Ecology*, **43**, 43–50.

Iponga, D.M., Milton, S.J. & Richardson, D.M. (2008) Superiority in competition for light: a crucial attribute defining the impact of the invasive alien tree *Schinus molle* (Anacardiaceae) in South African savanna. *Journal of Arid Environments*, **72**, 612–623.

Jackson, J. (2005) Is there a relationship between herbaceous species richness and buffel grass (*Cenchrus ciliaris*)? *Austral Ecology*, **30**, 505–517.

Jager, H., Tye, A. & Kowarik, I. (2007) Tree invasion in naturally treeless environments: impacts of quinine (*Cinchona pubescens*) trees on native vegetation in Galapagos. *Biological Conservation*, **140**, 297–307.

Johnson, N.C. & Wedin, D.A. (1997) Soil carbon, nutrients, and mycorrhizae during conversion of dry tropical forest to grassland. *Ecological Applications*, **7**, 171–182.

Kao-Kniffin, J. & Balser, T.C. (2008) Soil fertility and the impact of exotic invasion on microbial communities in Hawaiian forests. *Microbial Ecology*, **56**, 55–63.

Kappes, H., Lay, R. & Topp, W. (2007) Changes in different trophic levels of litter-dwelling macrofauna associated with giant knotweed invasion. *Ecosystems*, **10**, 734–744.

Keeley, J.E., Brennan, T. & Pfaff, A.H. (2008) Fire severity and ecosytem responses following crown fires in California shrublands. *Ecological Applications*, **18**, 1530–1546.

Keller, B.E. (2000) Plant diversity in *Lythrum*, *Phragmites*, and *Typha*-marshes, Massachusetts, USA. *Wetlands Ecology and Management*, **8**, 391–401.

Kennedy, T.A., Finlay, J.C. & Hobbie, S.E. (2005) Eradication of invasive *Tamarix ramosissima* along a desert stream increases native fish density. *Ecological Applications*, **15**, 2072–2083.

Kohli, R.K., Batish, D.R., Singh, H.P. & Dogra, K.S. (2006) Status, invasiveness and environmental threats of three tropical American invasive weeds (*Parthenium hysterophorus* L., *Ageratum conyzoides* L., *Lantana camara* L.) in India. *Biological Invasions*, **8**, 1501–1510.

Kourtev, P., Huang, W. & Ehrenfeld, J. (1999) Differences in earthworm densities and nitrogen dynamics in soils under exotic and native plant species. *Biological Invasions*, **1**, 237–245.

Kourtev, P.S., Ehrenfeld, J.G. & Haggblom, M. (2002) Exotic plant species alter the microbial community structure and function in the soil. *Ecology*, **83**, 3152–3166.

Koutika, L.S., Vanderhoeven, S., Chapuis-Lardy, L., Dassonville, N. & Meerts, P. (2007) Assessment of changes in soil organic matter after invasion by exotic plant species. *Biology and Fertility of Soils*, **44**, 331–341.

Kueffer, C., Schumacher, E., Fleischmann, K., Edwards, P.J. & Dietz, H. (2007) Strong below-ground competition shapes tree regeneration in invasive *Cinnamomum verum* forests. *Journal of Ecology*, **95**, 273–282.

Kurten, E.L., Snyder, C.P., Iwata, T. & Vitousek, P.M. (2008) *Morella cerifera* invasion and nitrogen cycling on a lowland Hawaiian lava flow. *Biological Invasions*, **10**, 19–24.

Kwiatkowska, A.J., Spalik, K., Michalak, E., Palinska, A. & Panufnik, D. (1997) Influence of the size and density of *Carpinus betulus* on the spatial distribution and rate of deletion of forest-floor species in thermophilous oak forest. *Plant Ecology*, **129**, 1–10.

Lambrinos, J.G. (2000) The impact of the invasive alien grass *Cortaderia jubata* (Lemoine): stapf on an endangered mediterranean-type shrubland in California. *Diversity and Distributions*, **6**, 217–231.

Lesica, P. & DeLuca, T.H. (2004) Is tamarisk allelopathic? *Plant and Soil*, **267**, 357–365.

Lesica, P. & Shelly, J.S. (1996) Competitive effects of *Centaurea maculosa* on the population dynamics of *Arabis fecunda*. *Bulletin of the Torrey Botanical Club*, **123**, 111–121.

Leslie, A.J. & Spotila, J.R. (2001) Alien plant threatens Nile crocodile (*Crocodylus niloticus*) breeding in Lake St. Lucia, South Africa. *Biological Conservation*, **98**, 347–355.

Ley, R.E. & D'Antonio, C.M. (1998) Exotic grass invasion alters potential rates of N fixation in Hawaiian woodlands. *Oecologia*, **113**, 179–187.

Liao, C.Z., Luo, Y.Q., Jiang, L.F., Zhou, X.H., Wu, X.W., Fang, C.M., Chen, J.K. & Li, B. (2007) Invasion of *Spartina alterniflora* enhanced ecosystem carbon and nitrogen stocks in the Yangtze Estuary, China. *Ecosystems*, **10**, 1351–1361.

Lindsay, E.A. & French, K. (2005) Litterfall and nitrogen cycling following invasion by *Chrysanthemoides monilifera* ssp. *rotundata* in coastal Australia. *Journal of Applied Ecology*, **42**, 556–566.

Lindsay, E.A. & French, K. (2006) The impact of the weed *Chrysanthemoides monilifera* ssp. *rotundata* on coastal leaf litter invertebrates. *Biological Invasions*, **8**, 177–192.

Litton, C.M., Sandquist, D.R. & Cordell, S. (2006) Effects of non-native grass invasion on aboveground carbon pools and tree population structure in a tropical dry forest of Hawaii. *Forest Ecology and Management*, **231**, 105–113.

Lloyd, J.D. & Martin, T.E. (2005) Reproductive success of chestnut-collared longspurs in native and exotic grassland. *The Condor*, **107**, 363–374.

Lopezaraiza-Mikel, M.E., Hayes, R.B., Whalley, M.R. & Memmott, J. (2007) The impact of an alien plant on a native plant-pollinator network: an experimental approach. *Ecology Letters*, **10**, 539–550.

Macdonald, I.A.W. & Frame, G.W. (1988) The invasion of introduced species into nature reserves in tropical savannas and dry woodlands. *Biological Conservation*, **44**, 67–93.

Maerz, J.C., Blossey, B. & Nuzzo, V. (2005) Green frogs show reduced foraging success in habitats invaded by Japanese knotweed. *Biodiversity and Conservation*, **14**, 2901–2911.

Maerz, J.C., Brown, C.J., Chapin, C.T. & Blossey, B. (2005) Can secondary compounds of an invasive plant affect larval amphibians? *Functional Ecology*, **19**, 970–975.

Marchante, E., Kjoller, A., Struwe, S. & Freitas, H. (2008) Invasive *Acacia longifolia* induce changes in the microbial catabolic diversity of sand dunes. *Soil Biology & Biochemistry*, **40**, 2563–2568.

Marchante, E., Kjoller, A., Struwe, S. & Freitas, H. (2008) Short- and long-term impacts of *Acacia longifolia* invasion on the belowground processes of a Mediterranean coastal dune ecosystem. *Applied Soil Ecology*, **40**, 210–217.

Martin, M.R., Tipping, P.W. & Sickman, J.O. (2009) Invasion by an exotic tree alters above and belowground ecosystem components. *Biological Invasions*, **11**, 1883–1894.

Martin, P.H. (1999) Norway maple (*Acer platanoides*) invasion of a natural forest stand: understory consequence and regeneration pattern. *Biological Invasions*, **1**, 215–222.

Mason, T.J. & French, K. (2008) Impacts of a woody invader vary in different vegetation communities. *Diversity and Distributions*, **14**, 829–838.

Mason, T.J., French, K. & Russell, K.G. (2007) Moderate impacts of plant invasion and management regimes in coastal hind dune seed banks. *Biological Conservation*, **134**, 428–439.

Mayer, P.M., Tunnell, S.J., Engle, D.M., Jorgensen, E.E. & Nunn, P. (2005) Invasive grass alters litter decomposition by influencing macrodetritivores. *Ecosystems*, **8**, 200–209.

McPherson, G.R., Wright, H.A. & Wester, D.B. (1988) Patterns of shrub invasion in semiarid Texas grasslands. *American Midland Naturalist*, **120**, 391–397.

Melgoza, G., Nowak, R.S. & Tausch, R.J. (1990) Soil-water exploitation after fire - competition between *Bromus tectorum* (Cheatgrass) and 2 native species. *Oecologia*, **83**, 7–13.

Meyerson, L., Saltonstall, K., Windham, L., Kiviat, E. & Findlay, S. (2000) A comparison of *Phragmites australis* in freshwater and brackish marsh environments in North America. *Wetlands Ecology and Management*, **8**, 89–103.

Meyerson, L.A., Chambers, R.M. & Vogt, K.A. (1999) The effects of *Phragmites* removal on nutrient pools in a freshwater tidal marsh ecosystem. *Biological Invasions*, **1**, 129–136.

Mgobozi, M.P., Somers, M.J. & Dippenaar-Schoeman, A.S. (2008) Spider responses to alien plant invasion: the effect of short- and long-term *Chromolaena odorata* invasion and management. *Journal of Applied Ecology*, **45**, 1189–1197.

Mills, J.E., Reinartz, J.A., Meyer, G.A. & Young, E.B. (2009) Exotic shrub invasion in an undisturbed wetland has little community-level effect over a 15-year period. *Biological Invasions*, **11**, 1803–1820.

Milton, S.J. & Siegfried, W.R. (1981) Above-ground biomass of Australian acacias in the southern Cape, South Africa. *Journal of South African Botany*, **47**, 701–716.

Mummey, D.L. & Rillig, M.C. (2006) The invasive plant species *Centaurea maculosa* alters arbuscular mycorrhizal fungal communities in the field. *Plant and Soil*, **288**, 81–90.

Munoz, A.A. & Cavieres, L.A. (2008) The presence of a showy invasive plant disrupts pollinator service and reproductive output in native alpine species only at high densities. *Journal of Ecology*, **96**, 459–467.

Muralli, K.S. & Setty, R.S. (2001) Effect of weeds *Lantana camara* and *Chromolaena odorata* growth on the species diversity, regeneration and stem density of tree and shrub layer in BRT sanctuary. *Current Science*, **80**, 675–678.

Musil, C.F. (1993) Effect of invasive Australian acacias on the regeneration, growth and nutrient chemistry of South-African lowland fynbos. *Journal of Applied Ecology*, **30**, 361–372.

Musil, C.F. & Midgley, G.F. (1990) The relative impact of invasive Australian acacias, fire and season on the soil chemical status of a sand plain lowland fynbos community. *South African Journal of Botany*, **56**, 419–427.

Nadkarni, N.M., Odion, D.C. & Garden, S.B.B. (1986) Effects of seedling an exotic grass *Lolium multiflorum* on native seedling regeneration following fire in a chaparral community. *Proceedings of the chaparral ecosystems research conference*, Report **62**, 115–121. California Water Resource Center, Santa Barbara.

Nilsson, C., Engelmark, O., Cory, J., Forsslund, A. & Carlborg, E. (2008) Differences in litter cover and understorey flora between stands of introduced lodgepole pine and native scots pine in Sweden. *Forest Ecology and Management*, **255**, 1900–1905.

Niu, H.B., Liu, W.X., Wan, F.H. & Liu, B. (2007) An invasive aster (*Ageratina adenophora*) invades and dominates forest understories in China: altered soil microbial communities facilitate the invader and inhibit natives. *Plant and Soil*, **294**, 73–85.

Ogle, S.M., Reiners, W.A. & Gerow, K.G. (2003) Impacts of exotic annual brome grasses (*Bromus* spp.) on ecosystem properties of northern mixed grass prairie. *American Midland Naturalist*, **149**, 46–58.

Orrock, J.L., Witter, M.S. & Reichman, O.J. (2008) Apparent competition with an exotic plant reduces native plant establishment. *Ecology*, **89**, 1168–1174.

Ortega, Y.K., McKelvey, K.S. & Six, D.L. (2006) Invasion of an exotic forb impacts reproductive success and site fidelity of a migratory songbird. *Oecologia*, **149**, 340–351.

Oswalt, C.M., Oswalt, S.N. & Clatterbuck, W.K. (2007) Effects of *Microstegium vimineum* (Trin.) A. Camus on native woody species density and diversity in a productive mixed-hardwood forest in Tennessee. *Forest Ecology and Management*, **242**, 727–732.

Otto, S., Groffman, P.M., Findlay, S.E.G. & Arreola, A.E. (1999) Invasive plant species and microbial processes in a tidal freshwater marsh. *Journal of Environmental Quality*, **28**, 1252–1257.

Paritsis, J. & Aizen, M.A. (2008) Effects of exotic conifer plantations on the biodiversity of understory plants, epigeal beetles and birds in *Nothofagus dombeyi* forests. *Forest Ecology and Management*, **255**, 1575–1583.

Parsons, J.J. (1972) Spread of African pasture grasses to the American tropics. *Journal of Range Management*, **25**, 12–17.

Petillon, J., Ysnel, F., Canard, A. & Lefeuvre, J.C. (2005) Impact of an invasive plant (*Elymus athericus*) on the conservation value of tidal salt marshes in western France and implications for management: responses of spider populations. *Biological Conservation*, **126**, 103–117.

Petsikos, C., Dalias, P. & Troumbis, A.Y. (2007) Effects of *Oxalis pes-caprae* L. invasion in olive groves. *Agriculture Ecosystems & Environment*, **120**, 325–329.

Pickart, A.J., Miller, L.M. & Duebendorfer, T.E. (1998) Yellow bush lupine invasion in northern California coastal dunes - I. Ecological impacts and manual restoration techniques. *Restoration Ecology*, **6**, 59–68.

Potts, D.L., Harpole, W.S., Goulden, M.L. & Suding, K.N. (2008) The impact of invasion and subsequent removal of an exotic thistle, *Cynara cardunculus*, on CO_2_ and H_2_O vapor exchange in a coastal California grassland. *Biological Invasions*, **10**, 1073–1084.

Pouchard, A., Garcia, R.A., Pena, E., Gonzalez, C., Cavieres, L.A. & Bustamante, R.O. (2008) Positive feedbacks between plant invasions and fire regimes: *Teline monspessulana* (L.) K. Koch (Fabaceae) in central Chile. *Biological Invasions*, **10**, 547–553.

Pritekel, C., Whittemore-Olson, A., Snow, N. & Moore, J.C. (2006) Impacts from invasive plant species and their control on the plant community and belowground ecosystem at Rocky Mountain National Park, USA. *Applied Soil Ecology*, **32**, 132–141.

Pyšek, P. & Pyšek, A. (1995) Invasion by *Heracleum mantegazzianum* in different habitats in the Czech Republic. *Journal of Vegetation Science*, **6**, 711–718.

Ramos, J.A. (1996) Introduction of exotic tree species as a threat to the Azores bullfinch population. *Journal of Applied Ecology*, **33**, 710–722.

Rand, T.A. & Louda, S.M. (2004) Exotic weed invasion increases the susceptibility of native plants attack by a biocontrol herbivore. *Ecology*, **85**, 1548–1554.

Reed, H.E., Seastedt, T.R. & Blair, J.M. (2005) Ecological consequences of C-4 grass invasion of a C-4 grassland: a dilemma for management. *Ecological Applications*, **15**, 1560–1569.

Reinhart, K.O., Greene, E. & Callaway, R.M. (2005) Effects of *Acer platanoides* invasion on understory plant communities and tree regeneration in the northern Rocky Mountains. *Ecography*, **28**, 573–582.

Rice, S.K., Westerman, B. & Federici, R. (2004) Impacts of the exotic, nitrogen-fixing black locust (*Robinia pseudoacacia*) on nitrogen-cycling in a pine-oak ecosystem. *Plant Ecology*, **174**, 97–107.

Richburg, J.A., Patterson, W.A. & Lowenstein, F. (2001) Effects of road salt and *Phragmites australis* invasion on the vegetation of a western Massachusetts calcareous lake-basin fen. *Wetlands*, **21**, 247–255.

Rodewald, A.D., Shustack, D.P. & Hitchcock, L.E. (2010) Exotic shrubs as ephemeral ecological traps for nesting birds. *Biological Invasions*, **12**, 33–39.

Rodgers, V.L., Wolfe, B.E., Werden, L.K. & Finzi, A.C. (2008) The invasive species *Alliaria petiolata* (garlic mustard) increases soil nutrient availability in northern hardwood-conifer forests. *Oecologia*, **157**, 459–471.

Rose, S. & Fairweather, P.G. (1997) Changes in floristic composition of urban bushland invaded by *Pittosporum undulatum* in northern Sydney, Australia. *Australian Journal of Botany*, **45**, 123–149.

Rossiter, N., Setterfield, S.A., Douglas, M.M., Hutley, L. & Cook, G. (2004) Exotic grass invasion in the tropical savanna of northern Australia: ecosystem consequences. *Weed management: Balancing people, planet, profit. Fourteenth Australian Weeds Conference* (eds B. M. [Sindel &](http://www.cabdirect.org:80/search.html?q=ed%3A"Sindel%2C+B.+M.")  S. B. [Johnson),](http://www.cabdirect.org:80/search.html?q=ed%3A"Johnson%2C+S.+B.") pp. 168–171. Wagga Wagga, New South Wales, Australia.

Rothstein, D.E., Vitousek, P.M. & Simmons, B.L. (2004) An exotic tree alters decomposition and nutrient cycling in a Hawaiian montane forest. *Ecosystems*, **7**, 805–814.

Saggar, S., McIntosh, P.D., Hedley, C.B. & Knicker, H. (1999) Changes in soil microbial biomass, metabolic quotient, and organic matter turnover under *Hieracium* (*H. pilosella* L.). *Biology and Fertility of Soils*, **30**, 232–238.

Sala, A., Smith, S.D. & Devitt, D.A. (1996) Water use by *Tamarix ramosissima* and associated phreatophytes in a Mojave Desert floodplain. *Ecological Applications*, **6**, 888–898.

Scheiman, D.M., Bollinger, E.K. & Johnson, D.H. (2003) Effects of leafy spurge infestation on grassland birds. *The Journal of Wildlife Management*, **67**, 115–121.

Schmidt, K.A. & Whelan, C.J. (1999) Effects of exotic *Lonicera* and *Rhamnus* on songbird nest predation. *Conservation Biology*, **13**, 1502–1506.

Schmidt, K.A., Nelis, L.C., Briggs, N. & Ostfeld, R.S. (2005) Invasive shrubs and songbird nesting success: effects of climate variability and predator abundance. *Ecological Applications*, **15**, 258–265.

Schooler, S.S., McEvoy, P.B. & Coombs, E.M. (2006) Negative per capita effects of purple loosestrife and reed canary grass on plant diversity of wetland communities. *Diversity and Distributions*, **12**, 351–363.

Schooler, S.S., McEvoy, P.B., Hammond, P. & Coombs, E.M. (2009) Negative per capita effects of two invasive plants, *Lythrum salicaria* and *Phalaris arundinacea*, on the moth diversity of wetland communities. *Bulletin of Entomological Research*, **99**, 229–243.

Siemens, T.J. & Blossey, B. (2007) An evaluation of mechanisms preventing growth and survival of two native species in invasive bohemian knotweed (*Fallopia* x *bohemica*, Polygonaceae). *American Journal of Botany*, **94**, 776–783.

Slobodchikoff, C.N. & Doyen, J.T. (1977) Effects of *Ammophila arenaria* on sand dune arthropod communities. *Ecology*, **58**, 1171–1175.

Smith, C.W. & Tunison, J.T. (1992) Fire and alien plants in Hawai’i: research and management implications for native ecosystems. *Alien plant invasions in native ecosystems of Hawaii: management and research* (eds C. P. Stone, C. W. Smith & J. T. Tunison), pp. 394–408. Cooperative National Park Resources Studies Unit University of Hawaii, Manoa.

Smoliak, S. & Dormaar, J.F. (1985) Productivity of Russian wildrye and crested wheatgrass and their effect on prairie soils. *Journal of Range Management*, **38**, 403–405.

Sperry, L.J., Belnap, J. & Evans, R.D. (2006) *Bromus tectorum* invasion alters nitrogen dynamics in an undisturbed arid grassland ecosystem. *Ecology*, **87**, 603–615.

St John, M.G., Wall, D.H. & Hunt, H.W. (2006) Are soil mite assemblages structured by the identity of native and invasive alien grasses? *Ecology*, **87**, 1314–1324.

Standish, R.J. (2004) Impact of an invasive clonal herb on epigaeic invertebrates in forest remnants in New Zealand. *Biological Conservation*, **116**, 49–58.

Standish, R.J., Robertson, A.W. & Williams, P.A. (2001) The impact of an invasive weed *Tradescantia fluminensis* on native forest regeneration. *Journal of Applied Ecology*, **38**, 1253–1263.

Standish, R.J., Williams, P.A., Robertson, A.W., Scott, N.A. & Hedderley, D.I. (2004) Invasion by a perennial herb increases decomposition rate and alters nutrient availability in warm temperate lowland forest remnants. *Biological Invasions*, **6**, 71–81.

Steenkamp, H.E. & Chown, S.L. (1996) Influence of dense stands of an exotic tree, *Prosopis glandulosa* Benson, on a savanna dung beetle (Coleoptera: Scarabeidae) assemblage in southern Africa. *Biological Conservation*, **78**, 305–311.

Steinaker, D.F. & Wilson, S.D. (2005) Belowground litter contributions to nitrogen cycling at a northern grassland-forest boundary. *Ecology*, **86**, 2825–2833.

Stewart, G. & Hull, A.C. (1949) Cheatgrass (*Bromus tectorum* L) - An ecologic intruder in Southern Idaho. *Ecology*, **30**, 58–74.

Stock, W.D., Wienand, K.T. & Baker, A.C. (1995) Impacts of invading N-2-fixing *Acacia* species on patterns of nutrient cycling in two Cape ecosystems: evidence from soil incubation studies and N-15 natural-abundance values. *Oecologia*, **101**, 375–382.

Suding, K.N., LeJeune, K.D. & Seastedt, T.R. (2004) Competitive impacts and responses of an invasive weed: dependencies on nitrogen and phosphorus availability. *Oecologia*, **141**, 526–535.

Svejcar, T. & Sheley, R. (2001) Nitrogen dynamics in perennial- and annual-dominated arid rangeland. *Journal of Arid Environments*, **47**, 33–46.

Thomas, C.D., Ng, D., Singer, M.C., Mallet, J.L.B., Parmesan, C. & Billington, H.L. (1987) Incorporation of a European weed into the diet of a North American herbivore. *Evolution*, **41**, 892–901.

Thorpe, A.S., Archer, V. & DeLuca, T.H. (2006) The invasive forb, *Centaurea maculosa*, increases phosphorus availability in Montana grasslands. *Applied Soil Ecology*, **32**, 118–122.

Toft, R.J., Harris, R.J. & Williams, P.A. (2001) Impacts of the weed *Tradescantia fluminensis* on insect communities in fragmented forests in New Zealand. *Biological Conservation*, **102**, 31–46.

Trammell, M.A. & Butler, J.L. (1995) Effects of exotic plants on native ungulate use of habitat. *Journal of Wildlife Management*, **59**, 808–816.

Treberg, M.A. & Husband, B.C. (1999) Relationship between the abundance of *Lythrum salicaria* (purple loosestrife) and plant species richness along the Bar River, Canada. *Wetlands*, **19**, 118–125.

Truscott, A.M., Palmer, S.C., Soulsby, C., Westaway, S. & Hulme, P.E. (2008) Consequences of invasion by the alien plant *Mimulus guttatus* on the-species composition and soil properties of riparian plant communities in Scotland. *Perspectives in Plant Ecology Evolution and Systematics*, **10**, 231–240.

Tunison, J.T., D‘Antonio, C.M. & Loh, R.K. (2001) Fire and invasive plants in Hawaii Volcanoes National Park. *Proceedings of the Invasive Species Workshop: the role of fire in the control and spread of invasive species* (eds K. E. M. Galley & T. P. Wilson), pp. 122–131. Miscellaneous Publication No. 11, Tall Timbers Research Station, Tallahassee, FL.

Turner, P.J., Scott, J.K. & Spafford, H. (2008) The ecological barriers to the recovery of bridal creeper (*Asparagus asparagoides* (L.) Druce) infested sites: impacts on vegetation and the potential increase in other exotic species. *Austral Ecology*, **33**, 713–722.

Tuttle, N., Beard, K. & Pitt, W. (2009) Invasive litter, not an invasive insectivore, determines invertebrate communities in Hawaiian forests. *Biological Invasions*, **11**, 845–855.

Valery, L., Bouchard, V. & Lefeuvre, J.C. (2004) Impact of the invasive native species *Elymus athericus* on carbon pools in a salt marsh. *Wetlands*, **24**, 268–276.

Valtonen, A., Jantunen, J. & Saarinen, K. (2006) Flora and lepidoptera fauna adversely affected by invasive *Lupinus polyphyllus* along road verges. *Biological Conservation*, **133**, 389–396.

van der Wal, R., Truscott, A.M., Pearce, I.S.K., Cole, L., Harris, M.P. & Wanless, S. (2008) Multiple anthropogenic changes cause biodiversity loss through plant invasion. *Global Change Biology*, **14**, 1428–1436.

Vanderhoeven, S., Dassonville, N. & Meerts, P. (2005) Increased topsoil mineral nutrient concentrations under exotic invasive plants in Belgium. *Plant and Soil*, **275**, 169–179.

Vanderhoeven, S., Dassonville, N., Chapuis-Lardy, L., Hayez M. & Meerts, P. (2006) Impact of the invasive alien plant *Solidago gigantea* primary productivity, plant nutrient content and soil mineral nutrient concentrations. *Plant and Soil*, **286**, 259–268.

Vanwilgen, B.W. & Richardson, D.M. (1985) The effects of alien shrub invasions on vegetation structure and fire behavior in South African fynbos shrublands: a simulation study. *Journal of Applied Ecology*, **22**, 955–966.

Vilà, M., Tessier, M., Suehs, C.M., Brundu, G., Carta, L., Galanidis, A., Lambdon, P., Manca, M., Medail, F., Moragues, E., Traveset, A., Troumbis, A.Y. & Hulme, P.E. (2006) Local and regional assessments of the impacts of plant invaders on vegetation structure and soil properties of Mediterranean islands. *Journal of Biogeography*, **33**, 853–861.

Vitousek, P.M. & Walker, L.R.; 1989. Biological invasion by *Myrica faya* in Hawaii: plant demography, nitrogen fixation, ecosystem effects. *Ecological Monographs*, **59**, 247–265.

Vitousek, P.M., Walker, L.R., Whiteaker, L.D., Muellerdombois, D. & Matson, P.A. (1987) Biological invasion by *Myrica faya* alters ecosystem development in Hawaii. *Science*, **238**, 802–804.

Vivrette, N.J. & Muller, C.H. (1977) Mechanism of invasion and dominance of coastal grassland by *Mesembryanthemum crystallinum*. *Ecological Monographs*, **47**, 301–318.

Von Holle, B., Joseph, K.A., Largay, E.F. & Lohnes, R.G. (2006) Facilitations between the introduced nitrogen-fixing tree, *Robinia pseudoacacia*, and nonnative plant species in the glacial outwash upland ecosystem of Cape Cod, MA. *Biodiversity and Conservation*, **15**, 2197–2215.

Walker, L.R. & Vitousek, P.M. (1991) An invader alters germination and growth of a native dominant tree in Hawaii. *Ecology*, **72**, 1449–1455.

Wardle, D.A., Nicholson, K.S. & Rahman, A. (1995) Ecological effects of the invasive weed species *Senecio jacobaea* L. (ragwort) in a New Zealand pasture. *Agriculture Ecosystems & Environment*, **56**, 19–28.

Wearne, L.J. & Morgan, J.W. (2004) Community-level changes in Australian subalpine vegetation following invasion by the non-native shrub *Cytisus scoparius*. *Journal of Vegetation Science*, **15**, 595–604.

Wedin, D.A. & Pastor, J. (1993) Nitrogen mineralization dynamics in grass monocultures. *Oecologia*, **96**, 186–192.

Wilcox, J. & Beck, C.W. (2007) Effects of *Ligustrum sinense* Lour. (Chinese privet) on abundance and diversity of songbirds and native plants in a southeastern nature preserve. *Southeastern Naturalist*, **6**, 535–550.

Wilkie, L., Cassis, G. & Gray, M. (2007) The effects on terrestrial arthropod communities of invasion of a coastal heath ecosystem by the exotic weed bitou bush (*Chrysanthemoides monilifera* ssp. *rotundata* L.). *Biological Invasions*, **9**, 477–498.

Williams, D.G. & Baruch, Z. (2000) African grass invasion in the Americas: ecosystem consequences and the role of ecophysiology. *Biological Invasions*, **2**, 123–140.

Williams, J.L. & Crone, E.E. (2006) The impact of invasive grasses on the population growth of *Anemone patens*, a long-lived native forb. *Ecology*, **87**, 3200–3208.

Williams, K., Westrick, L.J. & Williams, B.J. (2006) Effects of blackberry (*Rubus discolor*) invasion on oak population dynamics in a California savanna. *Forest Ecology and Management*, **228**, 187–196.

Williams, M.C. & Wardle, G.M. (2007) Pine and eucalypt litterfall in a pine-invaded eucalypt woodland: the role of fire and canopy cover. *Forest Ecology and Management*, **253**, 1–10.

Williams, P.A. & Karl, B.J. (1996) Fleshy fruits of indigenous and adventive plants in the diet of birds in forest remnants, Nelson, New Zealand. *New Zealand Journal of Ecology*, **20**, 127–145.

Windham, L. (2001) Comparison of biomass production and decomposition between *Phragmites australis* (common reed) and *Spartina patens* (salt hay grass) in brackish tidal marshes of New Jersey, USA. *Wetlands*, **21**, 179–188.

Windham, L. & Ehrenfeld, J.G. (2003) Net impact of a plant invasion on nitrogen-cycling processes within a brackish tidal marsh. *Ecological Applications*, **13**, 883–896.

Windham, L. & Lathrop, R.G. (1999) Effects of *Phragmites australis* (common reed) invasion on aboveground biomass and soil properties in brackish tidal marsh of the Mullica River, New Jersey. *Estuaries*, **22**, 927–935.

Witkowski, E.T.F. (1991) Effects of invasive alien acacias on nutrient cycling in the coastal lowlands of the Cape fynbos. *Journal of Applied Ecology*, **28**, 1–15.

Witkowski, E.T.F. (1991) Growth and competition between seedlings of *Protea repens* (L.) L. and the alien invasive, *Acacia saligna* (Labill) Wendl in relation to nutrient availability. *Functional Ecology*, **5**, 101–110.

Witkowski, E.T.F. & Mitchell, D.T. (1987) Variations in soil phosporus in the fynbos biome, South-Africa. *Journal of Ecology*, **75**, 1159–1171.

Wolf, J.J., Beatty, S.W. & Seastedt, T.R. (2004) Soil characteristics of Rocky Mountain National Park grasslands invaded by *Melilotus officinalis* and *M. alba*. *Journal of Biogeography*, **31**, 415–424.

Woods, K.D. (1993) Effects of invasion by *Lonicera tatarica* L. on herbs and tree seedlings in 4 New-England forests. *American Midland Naturalist*, **130**, 62–74.

Wootton, L.S., Halsey, S.D., Bevaart, K., McGough, A., Ondreicka, J. & Patel, P. (2005) When invasive species have benefits as well as costs: managing *Carex kobomugi* (Asiatic sand sedge) in New Jersey's coastal dunes. *Biological Invasions*, **7**, 1017–1027.

Wyckoff, P.H. & Webb, S.L. (1996) Understory influence of the invasive Norway maple (*Acer platanoides*). *Bulletin of the Torrey Botanical Club*, **123**, 197–205.

Yelenik, S.G., Stock, W.D. & Richardson, D.M. (2007) Functional group identity does not predict invader impacts: differential effects of nitrogen-fixing exotic plants on ecosystem function. *Biological Invasions*, **9**, 117–125.

Yoshida, K. & Oka, S. (2000) Impact of biological invasion of *Leucaena leucocephala* on successional pathway and species diversity of secondary forest on Hahajima Island, Ogasawara (Bonin) Islands, northwestern Pacific. *Japanese Journal of Ecology*, **50**, 111–119.

Young, J.A. & Evans, R.A. (1970) Invasion of medusahead into the Great Basin. *Weed Science*, **18**, 89–97.

Zalba, S.M., Cuevas, Y.A. & Boo, R.M. (2008) Invasion of *Pinus halepensis* Mill. following a wildfire in an Argentine grassland nature reserve. *Journal of Environmental Management*, **88**, 539–546.

Zedler, P.H., Gautier, C.R. & McMaster, G.S. (1983) Vegetation change in response to extreme events: the effect of a short interval between fires in California chaparral and coastal scrub. *Ecology*, **64**, 809–818.
