# Supplementary material for: A global assessment of invasive plant impacts on resident species, communities and ecosystems: the interaction of impact measures, invading species' traits and environment
Source: Glob Chang Biol. 2012 May;18(5):1725–37. doi: 10.1111/j.1365-2486.2011.02636.x (PMC3597245; doi:10.1111/j.1365-2486.2011.02636.x)
Supplement: Supplementary file 2 — Appendix S2. List of species that appeared in studies on impact of invasive plants included in the study. [file gcb0018-1725-SD2.doc]

**Appendix S2.** List of species that appeared in studies on impact of invasive plants included in the study. Number of cases in which impact response outcomes were recorded is given in parentheses.

Agavaceae: *Agave americana* (3), *Carpobrotus affine* (2), *Carpobrotus edulis* (6), *Mesembryanthemum crystallinum* (19)

Anacardiaceae: *Schinus molle* (2), *Schinus terebinthifolius* (1)

Apiaceae: *Anthriscus caucalis* (1), *Azorella monatha* (1), *Heracleum mantegazzianum* (36), *Imperatoria ostruthium* (2)

Araliaceae: *Hedera helix* (2)

Asclepiadaceae: *Orbea variegata* (6), *Vincetoxicum rossicum* (1)

Asteraceae: *Ageratina adenophora* (10), *Aster novi-belgii* (2), *Carduus nutans* (1), *Centaurea diffusa* (4), *Centaurea maculosa* (14), *Centaurea melitensis* (1), *Centaurea solstitialis* (2), *Cynara cardunculus* (1), *Delairea odorata* (9), *Helianthus tuberosus* (2), *Hieracium pilosella* (25), *Hypochoeris glabra* (1), *Chromolaena odorata* (9), *Chrysanthemoides monilifera* (37), *Mikania micrantha* (8), *Rudbeckia laciniata* (2), *Senecio jacobaea* (8), *Solidago canadensis* (7), *Solidago gigantea* (56), *Taraxacum officinale* (4),

Balsaminaceae: *Impatiens glandulifera* (21)

Berberidaceae: *Berberis thunbergii* (8)

Brassicaceae: *Alliaria petiolata* (11), *Brassica nigra* (4), *Lepidium latifolium* (18)

Cactaceae: *Opuntia stricta* (2)

Caprifoliaceae: *Lonicera japonica* (4), *Lonicera maackii* (17), *Lonicera tatarica* (3)

Commelinaceae: *Tradescantia fluminensis* (13)

Cyperaceae: *Carex kobomugi* (4)

Dipsacaceae: *Dipsacus sylvestris* (1)

Elaeagnaceae: *Eleagnus umbellata* (17)

Euphorbiaceae: *Euphorbia esula* (25), *Sapium sebiferum* (11)

Fabaceae: *Cytisus scoparius* (12), *Falcataria molluccana* (30), *Lespedeza cuneata* (4), *Lupinus arboreus* (4), *Lupinus luteus* (9), *Lupinus polyphyllus* (5), *Melilotus alba* (6), *Melilotus officinalis* (6), *Prosopis glandulosa* (4), *Prosopis velutina* (2), *Robinia pseudoacacia* (32), *Teline monspessulana* (1), *Trifolium pratense* (1), *Ulex europaeus* (3)

Fagaceae: *Carpinus betulus* (2), *Castanea crenata* (2), *Quercus acutissima* (2)

Geraniaceae: *Erodium cicutarium* (1), *Pelargonium capitatum* (20)

Chenopodiaceae: *Salsola kali* (1)

Juniperaceae: *Juniperus pinchotii* (3)

Lauraceae: *Cinnamomum verum* (2)

Liliaceae: *Asparagus asparagoides* (1)

Lythraceae: *Lythrum salicaria* (34),

Malvaceae: *Lavatera arborea* (2),

Mimosaceae: *Acacia cyclops* (13), *Acacia longifolia* (13), *Acacia saligna* (62), *Leucaena leucocephala* (2), *Mimosa pigra* (12)

Myricaceae: *Morella cerifera* (4), *Morella faya* (11)

Myrtaceae: *Melaleuca quinquenervia* (15), *Psidium catleianum* (1)

Oleaceae: *Fraxinus uhdei* (6), *Ligustrum lucidum* (2), *Ligustrum sinense* (2)

Oxalidaceae: *Oxalis pes-caprae* (8)

Pinaceae: *Pinus caribaea* (4), *Pinus contorta* (3), *Pinus halepensis* (1), *Pinus radiata* (15), *Pinus sylvestris* (6), *Pseudotsuga menziesii* (12)

Pittosporaceae: *Pittosporum undulatum* (2)

Plantaginaceae: *Plantago lanceolata* (3)

Poaceae: *Aegilops triuncialis* (15), *Agropyron cristatum* (32), *Agropyron repens* (6), *Agrostis stolonifera* (5), *Ammophila arenaria* (2), *Andropogon bladhii* (13), *Andropogon guayanus* (2), *Andropogon virginicus* (3), *Anthoxanthum odoratum* (1), *Arundo donax* (6), *Avena barbata* (1), *Bromus diandrus* (2), *Bromus hordeaceus* (1), *Bromus inermis* (7), *Bromus japonicus* (5), *Bromus madritensis* (2), *Bromus rubens* (2), *Bromus tectorum* (118), *Cenchrus ciliaris* (5), *Cortaderia jubata* (2), *Cortaderia selloana* (10), *Ehrharta calycina* (12), *Elymus athericus* (7), *Elymus junceus* (14), *Eragrostis lehmanniana* (2), *Festuca arundinacea* (2), *Holcus lanatus* (1), *Hordeum marinum* subsp. *gussonearum* (2), *Hymenachne amplexicaulis* (6), *Hyparrhenia rufa* (17), *Imperata cylindrica* (2), *Lolium multiflorum* (4), *Lolium perenne* (1), *Melinis minutiflora* (25), *Microstegium vimineum* (9), *Panicum maximum* (3), *Pennisetum clandestinum* (1), *Pennisetum polystachion* (6), *Pennisetum setaceum* (9), *Phalaris arundinacea* (4), *Phragmites australis* (41), *Poa pratensis* (11), *Schismus barbatus* (2), *Schizachyrium condensatum* (6), *Spartina alternifolia* (8), *Spartina anglica* (12), *Tainiatherum asperum* (1), *Urochloa mutica* (3)

Polygonaceae: *Fallopia ×bohemica* (5), *Fallopia japonica* (94), *Fallopia sachalinensis* (11), *Rumex alpinus* (2)

Proteaceae: *Hakea sericea* (1)

Rhamnaceae: *Rhamnus cathartica* (7), *Rhamnus frangula* (3)

Rosaceae: *Prunus serotina* (20), *Pyracantha angustifolia* (2), *Rosa multiflora* (2), *Rosa rugosa* (16), *Rubus discolor* (2)

Rubiaceae: *Cinchona pubescens* (4)

Salicaceae: *Populus tremuloides* (5), *Salix x rubens* (2)

Sapindaceae: *Acer platanoides* (11), *Koelreuteria elegans* (3)

Scrophulariaceae s. l.: *Mimulus guttatus* (8)

Simaroubaceae: *Ailanthus altissima* (13)

Tamaricaceae: *Tamarix ramosissima* (25)

Taxodiaceae: *Cryptomeria japonica* (1)

Typhaceae: *Typha x glauca* (12)

Verbenaceae: *Lantana camara* (13), *Stachytartheta jamaicensis* (1)

Zingiberaceae: *Hedychium gardnerianum* (7)
